# Prevalence of Bovine Viral Diarrhea Virus in Ovine and Caprine Flocks: A Global Systematic Review and Meta-Analysis

**DOI:** 10.3389/fvets.2021.703105

**Published:** 2021-11-19

**Authors:** Nai-Chao Diao, Zi-Yang Chen, Jun-Feng Shi, Qi Wang, Chen-Yan Sheng, Bao-Yi Ma, Yang Yang, Yu-Han Sun, Kun Shi, Rui Du

**Affiliations:** ^1^College of Chinese Medicine Materials, Jilin Agricultural University, Changchun, China; ^2^College of Animal Science and Technology, Jilin Agricultural University, Changchun, China; ^3^Laboratory of Production and Product Application of Sika Deer of Jilin Province, Jilin Agricultural University, Changchun, China; ^4^Key Laboratory of Animal Production, Product Quality and Security, Ministry of Education, Jilin Agricultural University, Changchun, China

**Keywords:** bovine viral diarrhea virus, ovine, caprine, prevalence, meta-analysis

## Abstract

**Background:** Bovine viral diarrhea virus (BVDV) is the causative agent of bovine viral diarrhea. It can infect cattle, sheep, pigs, and other animals, causing diarrhea, miscarriage, and stillbirth, among other symptoms, and it can result in huge economic losses to animal husbandry. There are reports on BVDV infection rates in sheep and goat herds from all over the world and this meta-analysis aimed to evaluate the prevalence of and risk factors for BVDV in sheep and goats.

**Results:** Using the data of 41,297 sheep and goats in 24 countries/regions to calculate a comprehensive prevalence rate for BVDV. The overall prevalence of BVDV infection in sheep and goats was estimated to be 8.6% (95% CI: 5.2–12.7) by immunological methods and 7.3% (95% CI: 2.7–13.7) by molecular methods. Analysis by national income level revealed that prevalence is higher in middle-income countries than in high-income countries (*P* < 0.05). The study also compared prevalence rates by species of BVDV, sampling year, and test species, but did not find significant differences.

**Conclusion:** This systematic review and meta-analysis is the first to determine the global prevalence of BVDV in ovine and caprine flocks. The prevalence of BVDV in sheep and goat populations varies from region to region, and the situation is not optimistic in some countries.

## Introduction

Bovine viral diarrhea virus (BVDV) is currently classified as the genus *Pestivirus* and family *Flaviviridae*, it is a single-stranded, positive-sense RNA virus (1). The natural host of BVDV is cattle, but it can also infect goats, sheep, piglets, and other domestic and wild ruminants through transiently infected individuals and persistently infected (PI) animals (2). BVDV infection in goats typically result in reproductive-system diseases, but survival in PI goats that can survive are rare (3). The main clinical symptoms of BVDV include decreased productivity and fecundity, slow fetal growth, diarrhea, respiratory symptoms, reproductive dysfunction (such as abortion, teratogenicity, fetal mummification, and stillbirth), and immune dysfunction, all of which can be complicated by infection (4–6). Two species of BVDV, i.e., BVDV-1 (Pestivirus A) and BVDV-2 (Pestivirus B), have been identified as bovine pestiviruses, together with the HoBi-like pestivirus (Pestivirus H) (7). Each species has multiple subgenomes: BVDV-1 can be divided into at least 21 subgenomes (1a-1u) and BVDV-2 can be divided into four subgenomes (2a-2d) (8). BVDV-1, BVDV-2, Border disease virus (BDV), and HoBi-like viruses have been identified from sheep and cattle (9). Cattle, sheep, and goats are the main domestic livestock across the world, and widespread infection with BVDV disease has caused huge economic losses to the livestock industry (10), with the cross-infection of a variety of animals making prevention and control of the disease difficult (11).

In economic terms, sheep and goats occupy an important position in the global livestock breeding industry. For example, in the Kyrgyz Republic (Kyrgyzstan), animal husbandry (especially sheep breeding) has always been the basis for supporting the livelihoods of local people. The contribution of agricultural production to the economy is vital (about 45% of total income) (12). Australia is the country with the largest number of sheep stock and is the largest exporter of wool in the world, widely known as the “country on the back of sheep.” Its developed sheep- and goat- farming industry is world-renowned and has been the lifeblood of the country's agricultural development for many years (13). Similarly, sheep breeding is an important source of income for many agricultural countries in the world (14). Recent studies have shown that BVDV infection of sheep and goats can cause diarrhea, miscarriage of female animals, stillbirth and teratogenesis (3, 15, 16). Researchers have found BVDV in infected sheep fetal tissue, proving that the virus can be transmitted through the sheep placental barrier (17). Goats infected with BVDV have higher neonatal morbidity and mortality (18, 19). BVDV infection can cause immunosuppression and lead to mixed infection and secondary infection by other pathogens, seriously impact the sustainable development of the global livestock industry (20, 21).

To the best of the authors' knowledge, no systematic assessment of the prevalence of BVDV infection in ovine and caprine flocks worldwide has been conducted. Understanding the epidemiology and epidemic dynamics of sheep and goat BVDV will help prevent and control disease caused by BVDV infection of sheep and goats and will help to evaluate the effectiveness of vaccination. Therefore, we conducted a systematic review and meta-analysis to estimate the prevalence of BVDV infection in sheep and goat populations worldwide. This article aims to comprehensively evaluate the prevalence of BVDV infection in sheep and goats by conducting a meta-analysis of articles that have been published around the world. Through this process, we evaluate the geographical distribution in terms of factors such as climate and altitude as well as the main factors affecting BVDV infection in sheep and goat herds to provide guidance on and suggestions for the prevention and control of BVDV.

## Materials and Methods

This systematic review and meta-analysis was performed strictly in accordance with the relevant requirements of the Preferred Reporting Items for Systematic Reviews and Meta-Analyses (PRISMA) Statement (22) (Supplementary Material 1).

### Literature Retrieval Strategy

A literature search in Chinese and English was conducted, searching Chinese Web of Knowledge (CNKI), the Wanfang database, the Chongqing VIP database, PubMed, Science Direct, Web of Science, and Springer Link.

In Pubmed, we built Search Formula A: (Diarrhea Viruses, Bovine Viral [Mesh] OR BVDV OR Diarrhea Virus, Bovine Viral OR Bovine Viral Diarrhea Viruses OR Bovine Diarrhea Virus OR Bovine Diarrhea Viruses OR Diarrhea Virus, Bovine OR Diarrhea Viruses, Bovine OR Virus, Bovine Diarrhea OR Viruses, Bovine Diarrhea OR Bovine Pestivirus OR Bovine Pestiviruses OR Pestiviruses, Bovine). We then built Search Formula B: (sheep [MeSH Terms] OR Ovis OR Dall Sheep OR Ovis dalli OR Sheep, Dall OR Goats [MeSH Terms] OR Goat OR Capra OR Capras OR Sheep, Domestic OR Domestic Sheep OR Ovis ammon aries OR Ovis aries OR Mouflon OR Mouflons OR Ovis gmelini musimon OR Ovis aries musimon OR Small ruminants [MeSH Terms]). Finally, we used the logical “AND” with the two formulas to identify items satisfying both Search Formula A AND Search Formula B: (Diarrhea Viruses, Bovine Viral [Mesh] OR BVDV OR Diarrhea Virus, Bovine Viral OR Bovine Viral Diarrhea Viruses OR Bovine Diarrhea Virus OR Bovine Diarrhea Viruses OR Diarrhea Virus, Bovine OR Diarrhea Viruses, Bovine OR Virus, Bovine Diarrhea OR Viruses, Bovine Diarrhea OR Bovine Pestivirus OR Bovine Pestiviruses OR Pestiviruses, Bovine) AND (sheep [MeSH Terms] OR Ovis OR Dall Sheep OR Ovis dalli OR Sheep, Dall OR Goats [MeSH Terms] OR Goat OR Capra OR Capras OR Sheep, Domestic OR Domestic Sheep OR Ovis ammon aries OR Ovis aries OR Mouflon OR Mouflons OR Ovis gmelini musimon OR Ovis aries musimon OR Small ruminants [MeSH Terms]).

In Science Direct, we built the Search Formula: (“Diarrhea Viruses, Bovine Viral” OR BVDV OR Pestivirus) AND (sheep OR goat OR small ruminants) AND (prevalence). In Web of Science, we built the Search Formula: (“Diarrhea Viruses, Bovine Viral” OR BVDV OR Pestivirus) AND (sheep OR goat OR small ruminants) AND (prevalence). In SpringerLink, we built Search Formula: (“Diarrhea Viruses, Bovine Viral” OR BVDV OR Pestivirus) AND (sheep OR goat OR small ruminants) AND (prevalence). In the CNKI database, we used the term “BVDV” + “Viral diarrhea” AND “sheep and goats” + “small ruminants” (The words were spelled in Chinese; the “+” represents the Boolean operator “OR”). In the Wanfang database, we used the terms (BVDV OR Viral diarrhea) AND (sheep and goats OR small ruminants) (in Chinese). The types of articles found in the Wanfang database were limited to “papers in journals, degree theses and conferences.” In the VIP Chinese Journal Database, we used the search formulas: (BVDV OR Viral diarrhea) AND (sheep and goats OR small ruminants) (The words were spelled in Chinese and “OR”). The search strategies and search restrictions are reported in Supplementary Material 2. Endnote (version X9.3.1) was used to catalog the articles retrieved.

### Inclusion and Exclusion Criteria

The following inclusion criteria were applied: (1) the research objects were sheep and goats; (2) sample size was >30; (3) research content included the number of sheep and goats infected with viruses; and (4) research reports were published in Chinese or English.

The following exclusion criteria were applied: (1) reviews; (2) duplicate publications or studies with similar data; (3) incomplete, unclear, or obviously erroneous data that could not be resolved by contacting the authors; (4) research on BVDV vaccination; (5) other species besides sheep and goats; (6) the type of sample collected for the study is a fecal sample (Most of the material collected in the study was diarrhea, which may overestimate the positive rate and is not sufficiently representative of the true infection status of the herd).

### Quality Assessment

The quality of the selected literature depends on the level of proposal evaluation, formulation, and evaluation methods (23). Quality was scored using a method with a maximum of five points assigned to each study. The studies were graded according to whether they detailed random sampling, inspection methods, sampling time, had a sample size greater than or equal to 200, or contained four or more risk factors, with one point awarded for each of these criteria met. According to this standard, we defined a score of 4–5 as high quality, 2–3 as middle quality, and 0–1 as low quality.

### Literature Screening and Data Extraction

The screening of the literature was carried out by four trained reviewers. First, preliminary screening of the title and abstract of the literature was carried out, then the full text was evaluated and the statistics examined. The authors of the original studies were not contacted for more information, and no unpublished data were used. Any disagreements between reviewers were resolved by Zi-Yang Chen, the study author.

We referred to a standardized data collection table to extract the data contained in the article (24). The recorded information was as follows: first author; publication year; sampling time; country; national income level (according to World Bank data available at https://data.worldbank.org.cn/indicator/NY.GNP.MKTP.CD?name_desc = false), with national income level divided into low-income, middle-income, and high-income; immunological method (ELISA and SNT); feeding mode (free-range agriculture and concentrated agriculture); BVDV species (BVDV-1 and BVDV-2); test species (sheep and goat); subject age (under 1 year old, 1–2 years old, and over 2 years old); sample classification (aborted fetuses and serum). Article quality level (low quality, middle quality, high quality) was also recorded.

Geographic area and geographic factors (geographic factor data provided by the National Oceanic and Atmospheric Administration National Environmental Information Center at https://gis.ncdc.noaa.gov/maps/ncei/ cdo/monthly) were also extracted, including the latitude and longitude, altitude, annual average precipitation, and average annual temperature of the sampling area. We grouped latitude according to south or north, depending on position in relation to the equator. North and south were further divided into groups for every 30-degree section. The longitude subgroup was divided into west and east, with the 180-degree sections from the prime meridian to east and west each subdivided into 30-degree section groups. Altitude was divided into three groups according to international standards: 1,500–3,500 meters (medium altitude), 3,500–5,300 meters (high altitude), and >5,300 meters (extreme altitude) (25). The average annual precipitation subcomponents were <200 mm and 200–400 mm. The average annual temperature was divided into subgroups for every 10°C. Extracting the geographical factors presented some problems. If there was no weather station in the sampling area, we chose the station closest to the sampling location. The precipitation and average annual temperature data provided by the station was not always complete, so we chose the year closest to the sampling year to calculate averages. If no sampling year was given, we only extracted the latitude, longitude, and altitude of the sampling point.

### Statistical Analysis

A systematic review and meta-analysis of the included studies was conducted to calculate the combined prevalence of BVDV infection in sheep and goats. A meta-analysis of the ratio was performed using R software (version 4.0.0). According to previous research, double-arcsine transformation (PFT) can be used to transform the observed rates to make them closer to (in line with) the normal distribution (26, 27). This part of the code is in Supplementary Material 3. The formula for PFT is as follows:

$$\begin{array}{l} \text{t}=\text{arcsin}\left(\text{sqrt}\left(\text{r}/\left(\text{n}+1\right)\right)\right)+\text{arcsin}\left(\text{sqrt}\left(\left(\text{r}+1\right)/\left(\text{n}+\;1\right)\right)\right)\\ \text{se}\left(\text{t}\right)=\text{sqrt}\left(1/\left(\text{n}+\;0.5\right)\right)\\ \text{p}=\;{\left(\text{sin}\left(\text{t}/2\right)\right)}^{2} \end{array}$$

Note: t: transformed prevalence; r = positive number; n = sample size; se = standard error.

To investigate whether the results have obvious heterogeneity, “meta” software package was used to calculate and prepare forest plots, using a random-effects model to predict heterogeneity (26, 28–30). The statistics *I*^2^ and Cochrane's Q (represented as χ^2^ and *P-*values) were used to assess variability. *I*^2^ < 50% represented low heterogeneity and *I*^2^ > 50% represented high heterogeneity, describing the percentage of difference between studies due to heterogeneity. *P* < 0.05 was considered statistically significant. Funnel plots, Egger's test and “trim and fill analysis” were used to detect publication bias, with publication bias and heterogeneity subjectively judged through funnel plot symmetry. A symmetrical graph was taken as representing an absence of publication bias and heterogeneity; asymmetry suggested publication bias and heterogeneity. Egger's test, based on the *P*-value, was used to assess publication bias. When *P* ≥ 0.05, the deviation was considered non-existent. *P* < 0.05 was taken as indicative of publication bias. The trim and fill analysis uses an iterative algorithm that removes a very small sample of studies from the positive side of the funnel plot (the side of the funnel plot with the most studies in the literature), recalculates the total effect size, and then gradually adds these originally removed studies to the formula and recalculates, repeating this until the funnel plot is left-right symmetric around the recalculated total effect size. A sensitivity analysis was conducted to further determine the stability of the results obtained. This was done by deleting one study at a time, merging the remaining studies, and judging the impact of the deleted study on the overall data by comparing the differences before and after.

The factors we investigated included sampling year (before 2000, 2001–2010, and after 2010), national income level (medium and high), feeding mode (free-range agriculture and concentrated agriculture), BVDV specie (BVDV-1 and BVDV-2), species tested (sheep and goats), age of test subjects (below 1 year old, 1–2 years old and over 2 years old), sample classification (aborted fetuses and serum), article quality level (low, middle, and high quality), latitude (0–30°N, 0–30°S, 30–60°N), longitude (0–30°E, 30–60°E, 60–90°E, 90–120°E, 120–150°E, 0–30°W, 30–60°W), altitude (<1,500 m, 1500–3500 m, 3500–5300 m), precipitation (<200 mm, 200–400 mm), and temperature (<0°C, 1–10°C, 10–20°C, 20–30°C).

## Results

### Literature Screening Process and Results

**Figure 1:**
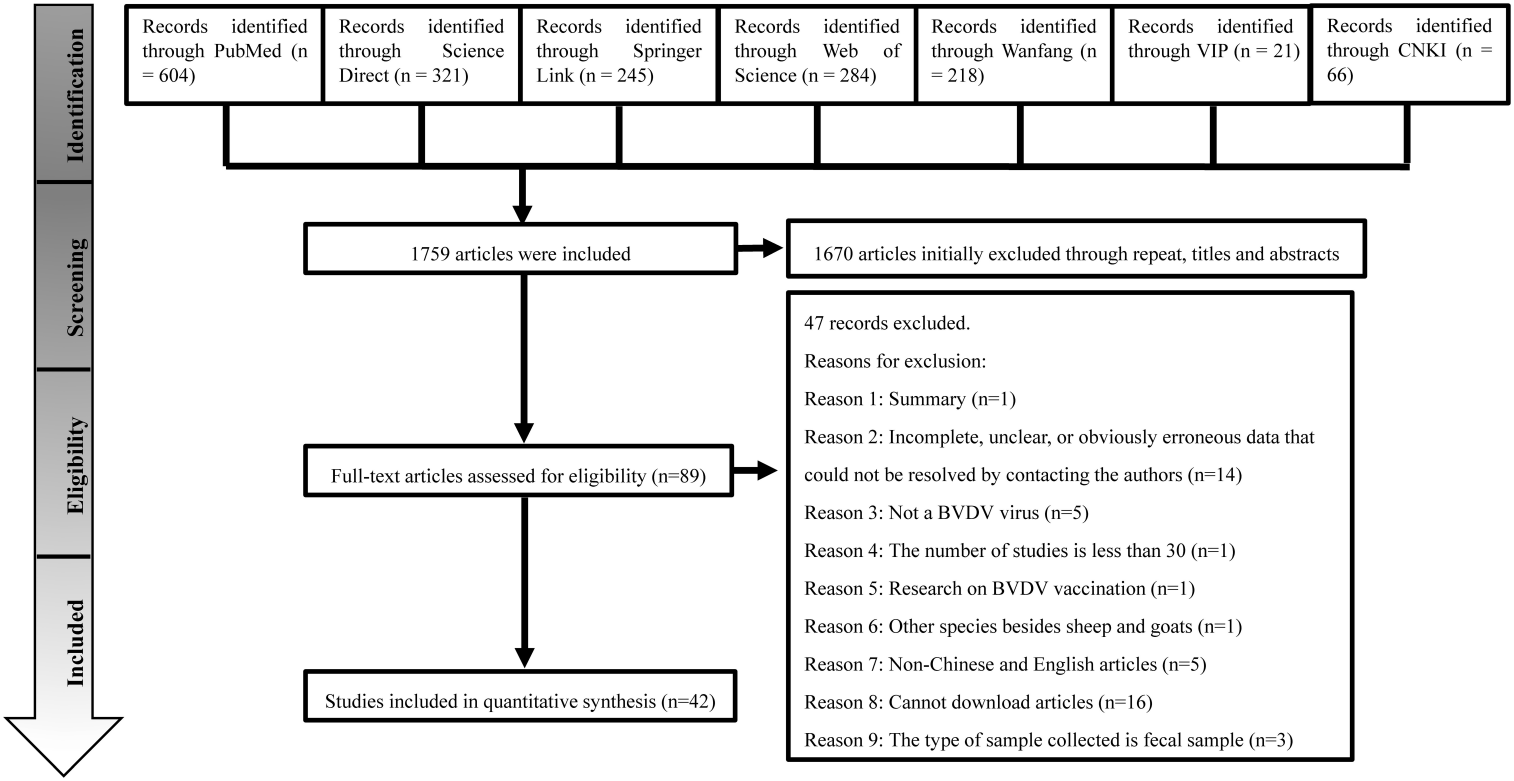
The selection process showing inclusion and exclusion of studies.

Figure 1 shows the PRISMA flow diagram. A total of 1,759 records were identified through database searching. Of these, 42 studies were eligible for the meta-analysis. Of the retrieved articles, 11 datasets were based on molecular methods, and 31 datasets were based on immunological methods.

Studies were identified from 24 countries worldwide, including four countries in Africa, two countries in South America, two countries in North America, seven countries in Europe, two countries in Oceania, and seven countries in Asia. Twenty-two studies were conducted in middle-income countries, and 20 studies were conducted in high-income countries. According to our quality rating scale, nine study articles were of middle quality (2–3 points), the remaining 32 were of high quality (four or five points), and only one article was of low quality (0–1 point) (Table 1).

**Table 1:** Included studies of BVDV infection in sheep and goats worldwide.

**Study ID**	**Sampling time**	**Income level**	**Country**	**Detection methods^**a**^**	**Positive samples/** **total samples**	**Quality score**	**Quality level**
**Africa**							
Hyera et al. (31)	1987	Middle	Tanzania	SNT	424/1564	5	High
Depner et al. (32)	1987–1989	Middle	Namibia	SNT	137/1736	5	High
Feknous et al. (33)	UN	Middle	Algeria	SNT	2/689	3	Middle
Lysholm et al. (34)	2016.09	Middle	Botswana	ELISA	0/100	4	High
**South America**							
Rosadio et al. (35)	UN	Middle	The Republic of Peru	SNT	1/34	2	Middle
Julia' et al. (36)	UN	High	Argentina	SNT	32/54	2	Middle
**Asia**							
Okur-Gumusova et al. (37)	UN	Middle	The Republic of Turkey	SNT	463/2444	4	High
Ataseven et al. (38)	2004.6–2005.2	Middle	The Republic of Turkey	SNT	83/275	5	High
Mishra et al. (39)	2004–2006	Middle	India	RT-PCR	2/562	5	High
Mishra et al. (40)	2004–2007	Middle	India	RT-PCR	8/1561	5	High
Yeşilbag and Gungor (41)	2004.4–2005.10	Middle	The Republic of Turkey	SNT	124/388	5	High
Yeşilbag et al. (42)	UN	Middle	The Republic of Turkey	SNT	0/137	2	Middle
Safarpoor Dehkordi (43)	2010	Middle	Iran	RT-PCR	167/967	4	High
Giangaspero et al. (44)	2007.9–2008.1	High	Japan	SNT	1/165	3	Middle
Li (45)	UN	Middle	China	SNT	12/35	3	Middle
Oem et al. (46)	2009.11–2011.8	High	Korea	ELISA	10/672	5	High
Kalaiyarasu et al. (47)	2005–2010	Middle	India	ELISA	187/569	4	High
Mao et al. (48)	2011–2013	Middle	China	RT-PCR	31/238	4	High
Mao et al. (49)	2011–2013	Middle	China	RT-PCR	29/236	4	High
Chen et al. (50)	2014.10–2016.6	Middle	China	RT-PCR	58/195	3	Middle
Bulut et al. (51)	2011–2015	Middle	The Republic of Turkey	RT-PCR	40/396	4	High
Deng et al. (52)	2013.3–2016.4	Middle	China	ELISA	38/217	4	High
Tamer et al. (53)	UN	Middle	The Republic of Turkey	SNT	101/543	4	High
Ma et al. (54)	2013.4–2014.3	Middle	China	ELISA	804/2187	5	High
Hidayat et al. (55)	2020.7–10	Middle	Indonesia	RT-PCR	2/46	4	High
**Europe**							
Løken et al. (56)	1980–1984	High	The Kingdom of Norway	SNT	166/3712	4	High
Løken (57)	1983–1984	High	The Kingdom of Norway	SNT	83/2335	4	High
Graham et al. (58)	1999.6–9	High	UK	SNT	14/918	4	High
O'Neill et al. (59)	2001.5.17–6.26	High	Republic of Ireland	SNT	39/1448	4	High
Krametter-Froetscher et al. (60)	UN	High	Austria	SNT	32/549	3	Middle
Danuser et al. (61)	2005–2006	High	Switzerland	ELISA	827/5562	5	High
Krametter-Froetscheet et al. (62)	2005–2006	High	Austria	RT-PCR	1/1196	4	High
Czopowicz et al. (63)	2007.6–7	High	Poland	ELISA	7/1060	5	High
Casaubon et al. (64)	2009–2011	High	Switzerland	ELISA	9/500	4	High
Decaro et al. (65)	2015–2016	High	Italy	RT-PCR	27/1231	4	High
Emma et al. (66)	2018.6–11	High	Northern Ireland	ELISA	56/3372	5	High
Potârniche et al. (67)	2014–2018	High	Poland	ELISA	7/910	5	High
**Oceania**							
Robinson (68)	UN	High	New Zealand	SNT	14/50	1	Low
Evans et al. (69)	UN	High	Australia	ELISA	0/875	4	High
Evans et al. (70)	2018.8–10	High	New Zealand	ELISA	17/270	4	High
**North America**							
Silveira et al. (9)	2015–2016	High	US	SNT	20/200	4	High
Lamontagne et al. (71)	UN	High	Canada	SNT	89/700	3	Middle

*Detection methods^a^: ELISA, enzyme-linked immunosorbent assay; RT-PCR, reverse transcription-polymerase chain reaction; SNT, serum neutralization test*.

### Evaluation of Publication Bias

**Figure 2:**
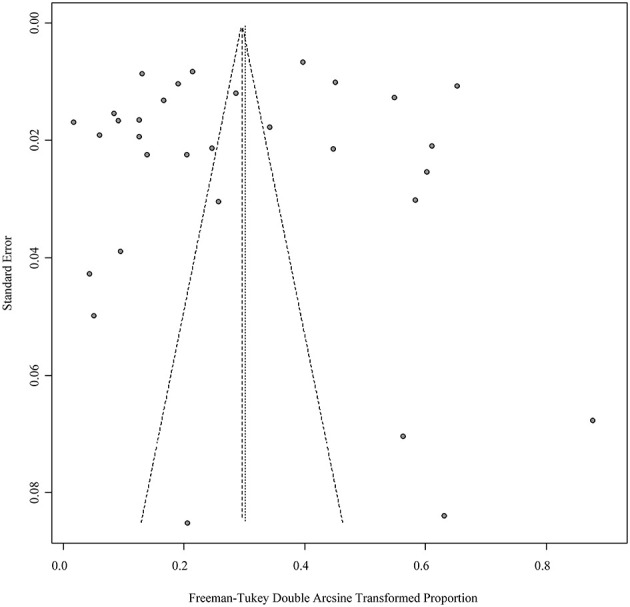
Publication bias of studies by funnel plot (Immunological methods).

**Figure 3:**
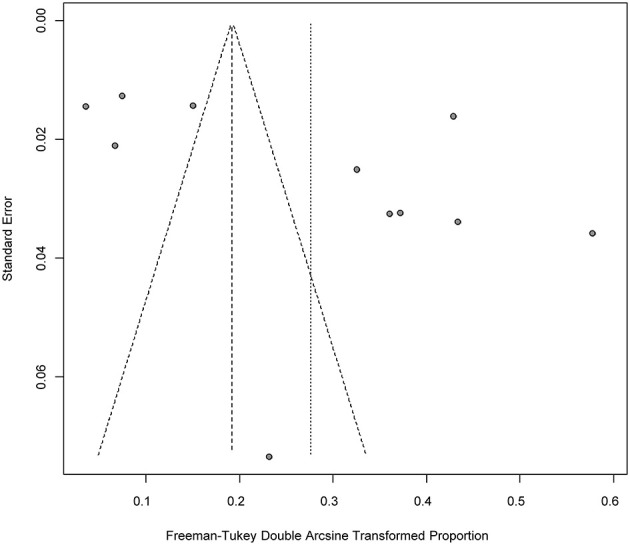
Publication bias of studies by funnel plot (Molecular methods).

**Figure 4:**
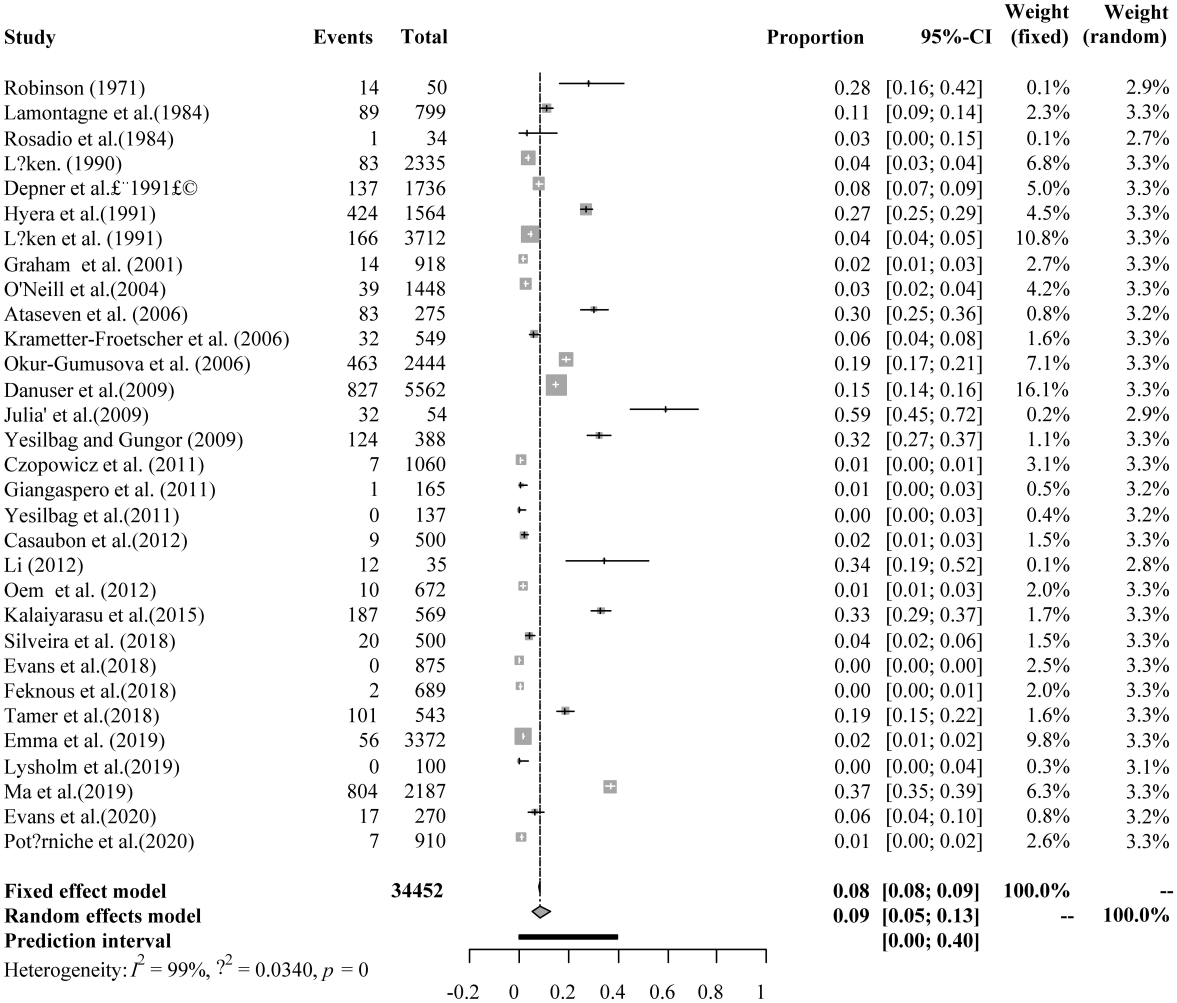
Random-effects meta-analysis of BVDV infection in sheep and goats (Immunological methods).

**Figure 5:**
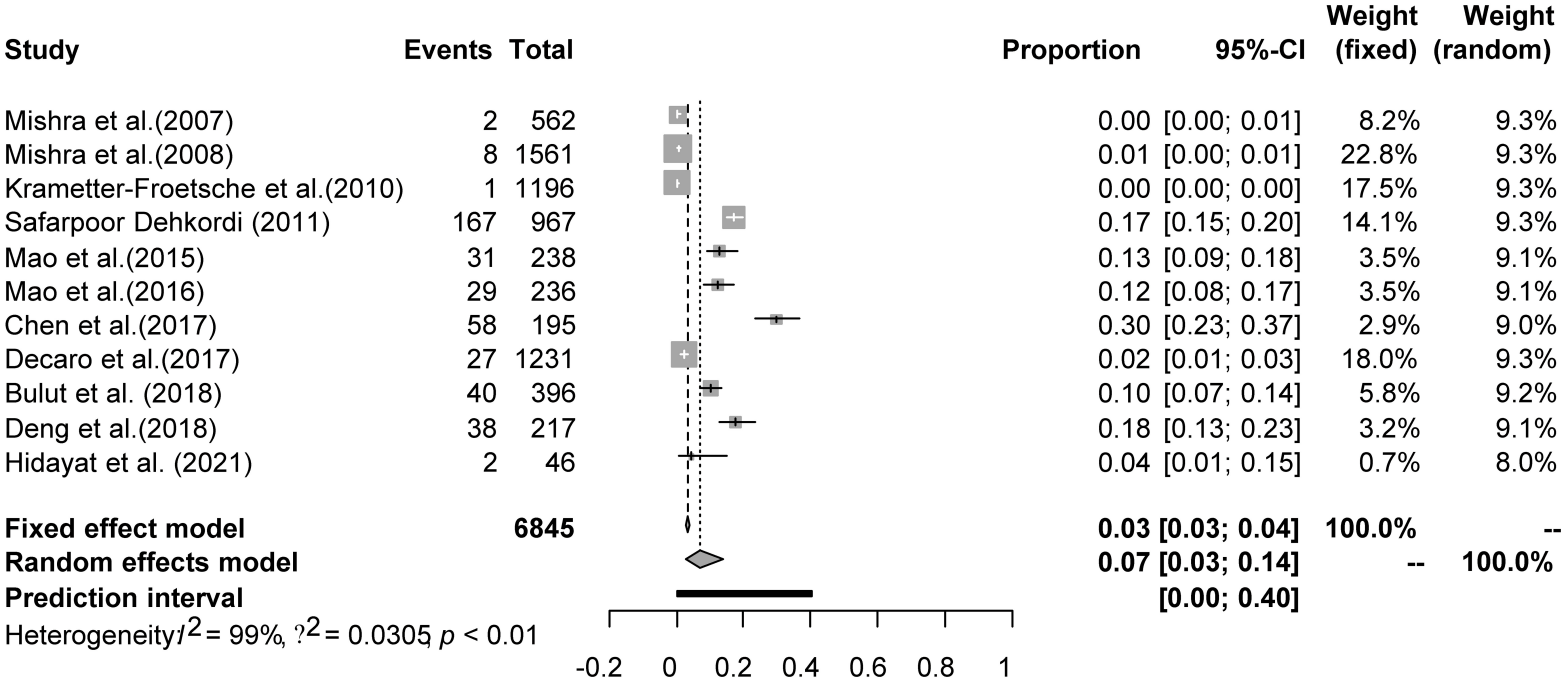
Random-effects meta-analysis of BVDV infection in sheep and goats (Molecular methods).

Four positive conversions were performed on the data (Tables 2, 3). The PFT conversion results were closer to the normal distribution, and we chose the combined results of the PFT conversion for meta-analysis. The extent of publication bias in the selected studies was evaluated and illustrated by funnel plots (Figures 2, 3). A random effects model (*I*^2^ = 99%) was used and visualized with forest plots (Figures 4, 5). According to the results of Egger's test (*P* = 0.7739 and *P* = 0.07888), we considered that the included articles were not biased for publication (Supplementary Figures 1, 2, Supplementary Tables 1, 2). The trim and fill analysis showed that the included studies had minimal sample size bias (Supplementary Figures 3, 4).

**Table 2:** Normal distribution test and conversion forms for the normal distribution of prevalence (Immunological methods).

**Conversion form**	***W***	***P***
PRAW	0.79631	4.414e-05
PLN	NaN	NA
PLOGIT	NaN	NA
PAS	0.91542	0.01788
PFT	0.91019	0.01307

*PRAW, original rate; PLN, logarithmic conversion; PLOGIT, logit transformation; PAS, arcsine transformation; PFT, double-arcsine transformation; NaN, meaningless number; NA, missing data*.

**Table 3:** Normal distribution test and conversion forms for the normal distribution of prevalence (Molecular methods).

**Conversion form**	***W***	***P***
PRAW	0.89584	0.1643
PLN	0.87417	0.08768
PLOGIT	0.8911	0.1435
PAS	0.93748	0.4914
PFT	0.94008	0.5214

*PRAW, original rate; PLN, logarithmic conversion; PLOGIT, logit transformation; PAS, arcsine transformation; PFT, double-arcsine transformation*.

### Sensitivity Analysis of the Included Literature

The sensitivity analysis (Supplementary Figures 5, 6) results showed that after removing one study, the reorganized results were consistent with the previous results, indicating that the systematic review and meta-analysis results were relatively stable and reliable.

### Meta-Analysis

#### Results Based on Immunological Methods

A total of 34,452 sheep and goats from 31 datasets were evaluated for detection of BVDV in their serum. The prevalence of BVDV infection in sheep and goats detected using immunological methods was 8.6% (95% CI: 5.2–12.7, 3,761/34,452; Table 4). In the regional subgroups, the prevalence for South America was 26.2% (95% CI: 0.0–87.8, 33/88; Table 5), which is higher than that of other regions. In terms of income-level subgroups, middle-income countries had the highest antibody prevalence of 14.9% (95% CI: 8.2–23.1, 2,338/10,701; Table 4).

**Table 4:** Association of different variables in the prevalence of BVDV infection in sheep and goats worldwide (Immunological methods).

	**No. studies**	**No. tested**	**No. positive**	**% (95% CI)^**b**^**	**Heterogeneity**			**Univariate meta-regression**	
					**χ2**	***P*-value**	***I^2^* (%)**	***P*-value**	**Coefficient (95% CI)**
**Sampling years**									
before 2000	5	10,265	824	7.2% (2.0–15.2)	666.12	<0.01	99.4%	0.664	0.038(−0.132–0.207)
2001–2010	8	10,070	1,100	8.7% (3.0–16.7)	900.27	<0.01	99.1%		
after 2011	8	8,597	1,093	6.4% (0.3–19.1)	2053.79	0.00	99.7%		
**Income level**									
Middle	13	10,701	2,338	14.9% (8.2–23.1)	1299.75	<0.01	99.1%	0.002	0.173(0.064–0.281)
High	18	23,751	1,423	4.8% (2.6–7.8)	1356.71	<0.01	98.7%		
**Method**									
ELISA	11	16,077	1,924	5.2% (0.8–12.9)	2696.34	0.00	99.6%	0.146	0.106(−0.040–0.249)
SNT	20	18,375	1,837	10.7% (6.6–15.6)	1674.50	0.00	98.9%		
**BVDV specie**									
BVDV-1	5	7,725	911	10.5% (2.6–21.4)	438.91	<0.01	99.1%	0.446	0.051(−0.068–0.417)
BVDV-2	2	743	9	4.0% (0.0–24.8)	20.45	<0.01	95.1%		
**Variety**									
Goat	17	9,743	785	8.7% (4.5–14.1)	1038.64	<0.01	98.5%	0.543	0.038(−0.083–0.159)
Sheep	22	24,709	2,976	11.4% (6.4–16.8)	3305.43	0.00	99.4%		
**Age**									
<1 year	3	1,772	180	13.0% (0.1–41.0)	318.52	<0.01	99.4%	0.014	0.275(0.056–0.494)
1–2 years	13	15,710	1,854	9.3% (4.3–16.0)	1667.66	0.00	99.3%		
>2 years	3	2,812	919	31.8% (24.2–40.0)	36.87	<0.01	94.6%		
**Feeding mode**									
Free-range agriculture	5	2,915	180	5.3% (1.6–10.8)	83.54	<0.01	95.2%	0.019	0.193(0.032–0.355)
Concentrated agriculture	13	13,778	2,591	17.9% (11.7–25.2)	1067.90	<0.01	98.9%		
**Quality level**									
Low	1	50	14	28.0% (16.3–41.4)	0.00	-	-	0.601	−0.040(−0.188–0.109)
Middle	8	2,462	169	8.4% (2.3–17.4)	264.72	<0.01	97.4%		
High	22	31,940	3,578	8.1% (4.3–12.8)	4037.40	0.00	99.5%		
Total	31	34,452	3,761	8.6% (5.2–12.7)	4373.38	0.000	99.3%		

*CI^b^, Confidence interval*.

*Young sheep or goat: <1 year*.

**Table 5:** Sub-group analysis of the prevalence of BVDV based on geographical factors (Immunological methods).

	**No. studies**	**No. tested**	**No.positive**	**% (95% CI)^**b**^**	**Heterogeneity**			**Univariate meta-regression**	
					**χ^2^**	***P*-value**	***I^2^* (%)**	***P*-value**	**Coefficient (95% CI)**
**Region**									
Africa	4	4,089	563	5.4% (0.0–19.7)	541.22	<0.01	99.4%	0.005	−0.182(-0.312–−0.054)
Asia	10	7,415	1,785	16.6% (8.5–26.8)	884.21	<0.01	99.0%		
Europe	10	20,366	1,240	3.1% (1.0–6.3)	1007.83	<0.01	99.1%		
Oceania	3	1,195	31	6.8% (0.0–24.3)	93.84	<0.01	97.9%		
South America	2	88	33	26.2% (0.0–87.8)	38.07	<0.01	97.4%		
North America	2	1,299	109	7.2% (1.8–15.7)	23.24	<0.01	95.7%		
**Latitude**									
0–30°N	1	569	187	32.9% (29.1–36.8)	0.00	-	-	0.092	−0.172(−0.373–0.028)
0–30°S	6	5,247	607	9.2% (1.2–23.1)	874.32	<0.01	99.4%		
30–60°N	9	13,447	1,936	20.2% (11.6–30.3)	1270.76	<0.01	99.4%		
**Longitude**									
0–30°E	4	7,198	986	11.0% (4.8–19.4)	181.27	<0.01	98.3%	0.205	0.190(0.104–0.484)
30–60°E	6	5,591	1,084	18.8% (8.9–31.1)	464.85	<0.01	98.9%		
60–90°E	1	569	187	32.9% (29.1–36.8)	0.00	-	-		
90–120°E	1	770	284	36.9% (33.5–40.3)	0.00	-	-		
120–150°E	2	910	12	9.2% (0.0–63.4)	51.31	<0.01	98.1%		
0–30°W	1	3,372	56	1.6% (1.3–2.1)	0.00	-	-		
30–60°W	2	853	121	32.2% (0.0–82.1)	58.59	<0.01	98.3%		
**Altitude**									
<1500 m	14	15,392	1,896	15.5% (8.8–23.7)	1960.24	0.00	99.3%	0.139	0.153(−0.050–0.355)
1500–3500 m	5	3,871	834	28.1% (13.5–45.6)	423.04	<0.01	99.1%		
**Annual average precipitation**									
<200 mm	5	7,215	779	11.4% (3.2–23.5)	652.43	<0.01	99.4%	0.280	0.219(−0.178–0.615)
200–400 mm	1	1,274	363	28.5% (26.1–31.0)	0.00	-	-		
**Annual average temperature**									
<0°C	1	1,274	363	28.5% (26.1–31.0)	0.00	-	-	0.104	−0.293(−0.647–0.060)
1–10°C	2	3,826	275	4.6% (0.1–14.5)	89.05	<0.01	98.9%		
10–20°C	4	1,914	210	20.3% (2.9–47.8)	354.77	<0.01	99.2%		
20–30°C	1	147	49	33.3% (25.9–41.2)	0.00	-	-		

*CI^b^, Confidence interval*.

As can be seen from Table 4, the prevalence is significantly different among age groups (*P* < 0.05), with the prevalence of 31.8% (95% CI: 24.2–40.0, 919/2,812) for animals over 2 years old being higher than that of the other age groups. There is no significant difference between subgroups by sampling year.

Furthermore, in terms of BVDV species, the prevalence of BVDV-1 was highest (10.5%, 95% CI: 2.6–21.4, 911/7,725). When comparing prevalence between goats and sheep, the prevalence in sheep (11.4%, 95% CI: 6.4–16.8, 2,976/24,709) was higher than that in goats (8.7%, 95% CI: 4.5–14.1, 785/9,743). In terms of feeding mode, the prevalence was higher in concentrated agriculture (17.9%, 95% CI: 11.7–25.2, 2,591/13,778) than in free-range agriculture (5.3%, 95% CI: 1.6–10.8, 180/2,915). According to quality of study, the prevalence was highest in studies of low quality (28.0%, 95% CI: 16.3–41.4, 14/50; Table 4). There were no significant differences between the geographic factor subgroups.

#### Results Based on Molecular Methods

A total of 6,845 sheep and goats from 11 datasets were evaluated for detection of BVDV in their aborted fetuses, and serum. Using the random-effects model, the prevalence of BVDV infection in sheep and goats detected using molecular methods was 7.3% (95% CI: 2.7–13.7, 403/6,845; Table 6). In the regional subgroups, the prevalence for Asia was 9.6% (95% CI: 3.3–18.6, 375/4,418; Table 7), which is higher than that for Europe (0.8%, 95% CI: 0.0–4.1, 28/2,427; Table 7). In terms of income level subgroups, middle-income countries had the highest viral prevalence at 9.6% (95% CI: 3.3–18.6, 375/4,418; Table 6).

**Table 6:** Association of different variables in the prevalence of BVDV infection in sheep and goats worldwide (Molecular methods).

**Variable/sub-groups**	**No. studies**	**No. tested**	**No. positive**	**% (95% CI)^**b**^**	**Heterogeneity**			**Univariate meta-regression**	
					**χ^2^**	***P*-value**	***I^2^* (%)**	***P*-value**	**Coefficient (95% CI)**
**Sampling years**									
2001–2010	4	4,286	178	2.2% (0.0–10.5)	415.63	<0.01	99.3%	0.070	0.200(−0.016–0.416)
after 2011	7	2,559	225	11.6% (4.9–20.5)	190.85	<0.01	96.9%		
**Income level**									
Middle	9	4,418	375	9.6% (3.3–18.6)	541.68	<0.01	98.5%	0.096	0.226(−0.040–0.493)
High	2	2,427	28	0.8% (0.0–4.1)	32.14	<0.01	96.9%		
**BVDV specie**									
BVDV-1	6	4,837	129	4.6% (1.2–10.2)	258.30	<0.01	98.1%	0.110	0.150(−0.002–0.301)
BVDV-2	3	3,354	16	0.4% (0.0–1.3)	15.22	<0.01	86.9%		
**Variety**									
Goat	8	3,342	202	7.0% (1.4–15.8)	415.84	<0.01	98.3%	0.990	0.001(−0.201–0.204)
Sheep	6	3,503	201	7.1% (1.3–16.4)	313.70	<0.01	98.4%		
**Feeding mode**									
Free-range agriculture	1	74	20	27.0% (17.5–37.8)	0.00	-	-	0.235	0.240(−0.637–0.156)
Concentrated agriculture	7	3,285	128	9.0% (2.7–18.5)	311.61	<0.01	98.1%		
**Sample**									
Aborted fetuses	2	1,363	207	13.6% (7.4–21.3)	12.15	<0.01	91.8%	0.212	0.140(−0.080–0.360)
Serum	8	5,244	165	5.3% (1.5–11.1)	363.47	<0.01	98.1%		
**Quality level**									
Middle	1	195	58	29.7% (23.5–36.4)	0.00	-	-	0.057	−0.332(−0.673–0.010)
High	10	6,650	345	5.8% (1.9–11.5)	600.40	<0.01	98.5%		
Total	11	6,845	403	7.3% (2.7–13.7)	720.35	<0.0001	98.6%		

*CI^b^, Confidence interval*.

*Young sheep or goat: <1 year*.

**Table 7:** Sub-group analysis of the prevalence of BVDV based on geographical factors (Molecular methods).

	**No. studies**	**No. tested**	**No. positive**	**% (95% CI)^**b**^**	**Heterogeneity**			**Univariate meta-regression**	
					**χ^2^**	***P*-value**	***I^2^* (%)**	***P*-value**	**Coefficient (95% CI)**
**Region**									
Asia	9	4,418	375	9.6% (3.3–18.6)	541.68	<0.01	98.5%	0.096	0.226(−0.040–0.493)
Europe	2	2,427	28	0.8% (0.0–4.1)	32.14	<0.01	96.9%		
**Latitude**									
0–30°S	1	46	2	4.4 (0.0–12.7)	0.00	-	-	0.785	0.057(−0.354–0.469)
30–60°N	5	3,236	155	8.0% (1.6–18.7)	294.07	<0.01	98.6%		
**Longitude**									
0–30°E	3	2,679	54	2.7% (0.0–8.7)	83.22	<0.01	97.6%	0.016	−0.215(−0.389–−0.041)
30–60°E	1	126	14	11.1% (6.2–17.3)	0.00	–	–		
90–120°E	3	367	73	13.8% (2.6–31.2)	28.72	<0.01	93.0%		
120–150°E	1	110	16	14.6% (8.5–21.8)	0.00	–	–		
**Altitude**									
<1500 m	4	1,730	62	5.5% (0.0–18.8)	146.42	<0.01	98.0	0.574	−0.117(−0.525–0.291)
1500–3500 m	1	1,231	27	2.2% (1.4–3.1)	0.00	–	–		
3500–5300 m	1	195	58	29.7% (23.5–36.4)	0.00	–	–		
**Annual average precipitation**									
<200 mm	3	1,613	81	9.2% (0.0–36.0)	232.74	<0.01	99.1%	0.635	0.161(−0.503–0.825)
200–400 mm	1	1,231	27	2.2% (1.4–3.1)	0.00	–	–		

*CI^b^, Confidence interval*.

As can be seen from Table 6, aborted fetuses samples had the highest prevalence at 13.6% (95% CI: 7.4–21.3, 207/1,363). The highest positivity rate in the sampling-year subgroup of was after 2011 at 11.6% (95% CI: 4.9–20.5, 225/2,559).

Furthermore, in terms of regarding BVDV species, the prevalence of BVDV-1 was highest (4.6%, 95% CI: 1.2–10.2, 129/4,837). When comparing viral prevalence between goats and sheep, the prevalence in sheep (7.1%, 95% CI: 1.3–16.4, 201/3,503) was higher than that in goats (7.0%, 95% CI: 1.4–15.8, 202/3,342). In terms of feeding mode, the prevalence was higher in free-range agriculture (27.0%, 95% CI: 17.5–37.8, 20/74) than in concentrated agriculture (9.0%, 95% CI: 2.7–18.5, 128/3,285). In terms of quality of study, the prevalence was highest in studies of middle quality (29.7%, 95% CI: 23.5–36.4, 58/195; Table 6). Among the geographic factor subgroups, the difference was significant in the longitude subgroup (*P* < 0.05), with the highest prevalence of 14.6% (95% CI: 8.5–21.8, 16/110; Table 7) in the 120–150°E group.

## Discussion

Sheep and goats are some of the earliest-domesticated animals. Because of their excellent reproduction performance, their products are distributed all over the world (72). Their meat, milk, wool, and leather play important roles in daily life, especially in low-income and developing countries (73). Cattle of all ages are susceptible to infection by BVDV, and its distribution is worldwide although some countries have recently eradicated the virus (OIE). Sheep and goats are similar to cattle, and they can be infected with BVDV at all ages (74). Acute infection usually manifests as intestinal and respiratory symptoms, and latent infection usually does not occur after recovery. Infection in the early stages of pregnancy can cause miscarriage or infertility, and in middle and late pregnancy, it can lead to fetal malformation or stillbirth. For infection in the first trimester of pregnancy, only a very small number (around 1–2%, cattle; OIE) of fetuses survive and form PI animals, which remain PI for life and are usually seronegative, presenting serious problems for the prevention and control of the virus (75). Therefore, detailed knowledge of the epidemiological status of BVDV has become crucial for the development of effective prevention measures in sheep and goats.

We searched all the articles on the epidemiology of sheep and goat BVDV from 1971 to 2021. Our meta-analysis finally considered 42 documents covering six continents and 24 countries and a total of 41,297 sheep and goats. Sheep and goats are widely distributed around the world, and both often coexist in the same farm, regardless of the fact that intensive and free-range farming often intersects geographically (76). In order to pursue quality increase, other species are often introduced, increasing the transmission of the disease (34, 77). In addition, the use of public pastoral areas, as well as the cross-working of breeding or technical personnel methods can contribute to the spread of BVDV, so we have analyzed and will discuss sheep and goats together. There are two main methods for detecting the prevalence of BVDV, namely immunological and molecular methods. In the studies, we considered 31 were based on immunological methods and 11 papers were based on molecular methods. In our statistical analysis, the total positive rate of immunological methods was 8.6% (95% CI: 5.2–12.7) and the total positive rate of molecular methods was 7.3% (95% CI: 2.7–13.7). Thus, the results of the two were essentially the same.

The immunological methods considered in this study are ELISA and SNT. The molecular method is RT–PCR. RT–PCR has high sensitivity and specificity. It is not affected by maternal antibodies, but may present false positives due to cross-contamination (78). Immunological and molecular methods have their advantages and disadvantages. In terms of detection sensitivity and accuracy, immunological methods cannot be compared with molecular methods (43, 79), but immunological methods have advantages over molecular methods in terms of batch detection and ease of operation. In terms of testing costs, immunological methods are more economical. In general, for clinical sample testing, immunological methods are recommended for large-scale screening in concert with, the use of molecular methods for retesting suspected or uncertain samples, ensuring accuracy.

The incidence of BVDV in sheep and goat populations may be related to management measures (80). Among the continental subpopulations, the prevalence of BVDV in Asian sheep and goats is relatively high. For Asia, the prevalence for immunological testing is 16.6% (95% CI: 8.5–26.8), and the prevalence for molecular testing is 9.6% (95% CI: 3.3–18.6), which are much higher than the corresponding values for Europe, where the prevalence for immunological testing was 3.1% (95% CI: 1.0–6.3) and the prevalence for molecular testing was 0.8% (95% CI: 0.0–4.1) (Tables 5, 7). According to income–level subgroup analysis, the prevalence of BVDV in sheep and goat herds in middle-income countries is higher than that in high-income countries, which is consistent with the results for the continental subgroups. However, there are several abnormal data revealed by the analysis. For example, the immunological prevalence rate in South America is 26.2% (95% CI: 0.0–87.8) and the immunological prevalence rate in Argentina is 59.3% (95% CI: 45.8–72.1). The actual epidemic situation in the region still needs more epidemiological analysis to support this data. It is noteworthy that Australia, a large agricultural country, has a BVDV prevalence rate close to zero although Australia has not yet introduced an elimination plan (81). This shows that scientifically informed breeding and effective management systems are closely related to animal health.

We analyzed the data in terms of sampling–year subgroup, and the prevalence rate of BVDV in sheep and goat herds after 2011 was 11.6% (95% CI: 4.9–20.5) (Table 6). The decrease in prevalence from 2001 to 2010 may be related to programs initiated by certain European countries to prevent BVDV in livestock. For example, Austria launched a mandatory BVDV control plan in August 2004 (82), Switzerland has implemented a mandatory eradication plan since 2008 (61, 83). In the present study, we divided the breeding methods into free-range and intensive agriculture. The positive rate for immunological methods in intensive agriculture was 17.9% (95% CI: 11.7–25.2), which is higher than that of free-range agriculture at 5.3% (95% CI: 1.6–10.8). However, the results of molecular methods contradict those of immunological methods. The positive rate for intensive agriculture was 9.0% (95% CI: 2.7–18.5), which is lower than the positive rate for free-range agriculture, at 27.0% (95% CI: 17.5–37.8) (Table 6). The main reason for this result is that molecular methods use a relatively small sampling volume and have a certain pertinence.

Our meta-analysis also looked at geographical factors. We did not find significant differences in BVDV prevalence by latitude, altitude, average annual precipitation, or mean annual temperature (*P* > 0.05; Tables 5, 7). The BVDV virus prevalence was lower in sheep and goat flocks in the 0–30°E longitude region (Table 7). According to the regional subgroup analysis, BVDV prevalence was not high in European countries, which corresponds to the results for longitude. Although BVDV prevalence in Europe was low, the study data suggest that the European sheep and goat population is not insignificant and that many countries with low prevalence are at risk of importing the virus. Therefore, adequate disease prevention and good management and control are needed to ensure imported livestock do not introduce disease (84).

BVDV is further classified into two species, BVDV-1 and BVDV-2. In our subgroup study, the prevalence for BVDV-1 was higher than that for BVDV-2 (Tables 4, 6). BVDV-2 virus is understood to have been originally isolated from a serious outbreak of hemorrhagic fever syndrome in cattle. The virus then spread to the European continent through contaminated fetal bovine serum or other biological products. The propagation rate of BVDV-2 is lower than that of BVDV-1 (60, 85). In Krametter-Froetscher et al. (86), the main pestivirus in Austrian sheep and goat flocks was found to be BVDV-1. An analysis by the same author reported in another article showed that BVDV-1 comprised the main genetic population (87). Mishra et al. (39, 40, 88) conducted research on plague virus infections in Indian sheep and goat flocks and found that BVDV-1 was widespread. BVDV-1 is the most common BVDV species in Africa (8). In terms of virulence, BVDV-2 is more pathogenic than BVDV-1 (89). Under natural conditions, these two species can cause double infection (90), thereby enhancing the toxicity of the virus. Research reports indicate that the virulence of species or strains is a factor in economic losses (91). Therefore, care should be taken to avoid the harm caused by mixed infection with BVDV-1 and BVDV-2 in the process of feeding and management.

In the age subgroup, we found that BVDV prevalence was highest in flocks of sheep and goats older than 2 years, and it was significantly higher than in flocks of sheep and goats under 1 year or 1–2 years old (*P* < 0.05, Table 4). The wide range of movement of adult sheep and the opportunities for contact with other species increase the risk of contracting the disease, which may also explain the high rate of positivity in adult sheep (61, 92, 93). Thus, decreasing movement between species and timely isolation of positive sheep and goats is essential for controlling BVDV disease and transmission.

There may be some limitations in our systematic review and meta-analysis, but these limitations are inevitable. First, although we retrieved a large number of papers using various retrieval methods, many of them were ineligible for inclusion. Second, there were very few reports from some countries and regions, or studies had small sample sizes. The actual epidemic situation in some surveyed countries and regions may not have been truly represented. Third, the quality of the included studies was uneven, meaning that the studies could not fully reflect the prevalence of sheep and goat BVDV in all areas, with more testing needed. Fourth, because of incomplete statistical information in the included articles, information was insufficient for complete analysis. For example, data such as season and gender were not analyzed in our systematic review and meta-analysis. Last, we only included research reports written in English and Chinese, which may have led to research in other languages being ignored, thereby affecting the results.

## Conclusion

In conclusion, despite their limitations, systematic reviews and meta-analysis studies provide a comprehensive overview of global antibody and virus prevalence in sheep and goats. We found a relatively high chance of infection in middle-income level countries and recommend better management of environmental health to reduce the risk of BVDV infection. The results of our study also indicate that ongoing surveillance of sheep and goats as well as ongoing implementation of integrated improvement strategy measures should be conducted. Furthermore, we recommend that additional surveys be conducted to further clarify the prevalence of BVDV worldwide.

## Data Availability Statement

The original contributions presented in the study are included in the article/Supplementary Material, further inquiries can be directed to the corresponding authors.

## Author Contributions

KS and RD conceptualized the study, with funding provided by RD. Data extraction was performed by Z-YC, Y-HS, YY, and B-YM. The database was established by Z-YC, and data analysis carried out by QW. N-CD wrote the original draft of the article, and Z-YC, J-FS, and C-YS reviewed and edited it. All authors contributed to the manuscript editing and approved the final manuscript.

## Funding

This work was supported by the National Natural Science Foundation of China (No. 31672577), Jilin Science &Technology Development Plan (20190301004NY), and the Scientific Research Planning Project of Jilin Provincial Department of Education (JJKH20200364KJ).

## Conflict of Interest

The authors declare that the research was conducted in the absence of any commercial or financial relationships that could be construed as a potential conflict of interest.

## Publisher's Note

All claims expressed in this article are solely those of the authors and do not necessarily represent those of their affiliated organizations, or those of the publisher, the editors and the reviewers. Any product that may be evaluated in this article, or claim that may be made by its manufacturer, is not guaranteed or endorsed by the publisher.

## Abbreviations

BDV: Border disease virus
ELISA: Enzyme-linked immunosorbent assay
SNT: Serum neutralization test
RT-PCR: Reverse transcription-polymerase chain reaction
OIE: World Organisation for Animal Health.

## References

[1] Schaut RG Ridpath JF Sacco RE. Bovine viral diarrhea virus type 2 impairs macrophage responsiveness to toll-like receptor ligation with the exception of toll-like receptor 7. PLoS ONE. (2016) 11:e0159491. 10.1371/journal.pone.0159491 27420479 PMC4946783

[2] Ridpath JF. Bovine viral diarrhea virus: global status. Vet Clin North Am Food Anim Pract. (2010) 26:105–21. 10.1016/j.cvfa.2009.10.007 20117546

[3] Passler T Riddell KP Edmondson MA Chamorro MF Neill JD Brodersen BW. Experimental infection of pregnant goats with bovine viral diarrhea virus (BVDV) 1 or 2. Vet Res. (2014) 45:38. 10.1186/1297-9716-45-38 24708266 PMC3994200

[4] Sprecher DJ Baker JC Holland RE Yamini B. An outbreak of fetal and neonatal losses associated with the diagnosis of bovine viral diarrhea virus. Theriogenology. (1991) 36:597–606. 10.1016/0093-691X(91)90397-V 16727029

[5] McGowan MR Kirkland PD Richards SG Littlejohns IR. Increased reproductive losses in cattle infected with bovine pestivirus around the time of insemination. Vet Rec. (1993) 133:39–43. 10.1136/vr.133.2.39 8212473

[6] Lanyon SR Hill FI Reichel MP Brownlie J. Bovine viral diarrhoea: pathogenesis diagnosis. Vet J. (2014) 199:201–9. 10.1016/j.tvjl.2013.07.024 24053990

[7] Decaro N. HoBi-Like pestivirus and reproductive disorders. Front Vet Sci. (2020) 7:622447. 10.3389/fvets.2020.622447 33415134 PMC7782308

[8] Yeşilbag K Alpay G Becher P. Variability and global distribution of subgenotypes of bovine viral diarrhea virus. Viruses. (2017) 9:128. 10.3390/v9060128 28587150 PMC5490805

[9] Silveira S Falkenberg SM Elderbrook MJ Sondgeroth KS Dassanayake RP Neill JD. Serological survey for antibodies against pestiviruses in Wyoming domestic sheep. Vet Microbiol. (2018) 219:96–9. 10.1016/j.vetmic.2018.04.019 29778211

[10] Ferreira AM Bislev SL Bendixen E Almeida AM. The mammary gland in domestic ruminants: a systems biology perspective. J Proteomics. (2013) 94:110–23. 10.1016/j.jprot.2013.09.012 24076120

[11] Houe H. Epidemiological features and economical importance of bovine virus diarrhoea virus (BVDV) infections. Vet Microbiol. (1999) 64:89–107. 10.1016/S0378-1135(98)00262-4 10028165

[12] Goletti F Chabot P. Food policy research for improving the reform of agricultural input and output markets in Central Asia. Food Policy. (2000) 25:661–79. 10.1016/S0306-9192(00)00036-1

[13] Cashin P Mcdermott CJ. ‘Riding on the sheep's back’: examining Australia's dependence on wool exports. Economic Record. (2010) 78:249–63. 10.1111/1475-4932.00055

[14] Pulina G Milán MJ Lavín MP Theodoridis A Morin E Capote J. Invited review: current production trends, farm structures, and economics of the dairy sheep and goat sectors. J Dairy Sci. (2018) 101:6715–29. 10.3168/jds.2017-14015 29859690

[15] Mao L Li WL Yan SH Zhao YQ Zhang ZB Jiang J. Isolation and identification of bovine viral diarrhea virus from goats. Chin Vet Sci. (2013) 43:684–8. 10.16656/j.issn.1673-4696.2013.07.002. 26992733

[16] Xu FF. Diagnosis of sheep infected with bovine viral diarrhea. CHIN J Anim husbandry Vet Med. (2016) 32:172. Available online at: https://kns.cnki.net/kcms/detail/detail.aspx?dbcode=CJFD&dbname=CJFDLAST2016&filename=ZXWA201606163&v=TTIeKRj6F6yN8rM4D%25mmd2F%25mmd2BUcwiagR9jIjvk51kWWBtJVkYYIzW

[17] Swasdipan S Bielefeldt-Ohmann H Phillips N Kirkland PD McGowan MR. Rapid transplacental infection with bovine pestivirus following intranasal inoculation of ewes in early pregnancy. Vet Pathol. (2001) 38:275–80. 10.1354/vp.38-3-275 11355657

[18] Pratelli A Martella V Cirone F Buonavoglia D Elia G Tempesta M. Genomic characterization of pestiviruses isolated from lambs and kids in southern Italy. J Virol Methods. (2001) 94:81–5. 10.1016/S0166-0934(01)00277-4 11337042

[19] Kim IJ Hyun BH Shin JH Lee KK Lee KW Cho KO. Identification of bovine viral diarrhea virus type 2 in Korean native goat (Capra hircus). Virus Res. (2006) 121:103–6. 10.1016/j.virusres.2006.04.008 16766076

[20] Baker JC. The clinical manifestations of bovine viral diarrhea infection. Vet Clin North Am Food Anim Pract. (1995) 11:425–45. 10.1016/S0749-0720(15)30460-6 8581856

[21] Braun U Bachofen C Schenk B Hässig M Peterhans E. Investigation of border disease and bovine virus diarrhoea in sheep from 76 mixed cattle and sheep farms in eastern Switzerland. Schweiz Arch Tierheilkd. (2013) 155:293–8. 10.1024/0036-7281/a000460 23644292

[22] Moher D Shamseer L Clarke M Ghersi D Liberati A Petticrew M. Preferred reporting items for systematic review and meta-analysis protocols (PRISMA-P) 2015 statement. Syst Rev. (2015) 4:1. 10.1186/2046-4053-4-1 25554246 PMC4320440

[23] Atkins D Best D Briss PA Eccles M Falck-Ytter Y Flottorp S. Grading quality of evidence and strength of recommendations. BMJ. (2004) 328:1490. 10.1136/bmj.328.7454.1490 15205295 PMC428525

[24] Ni H-B Gong QL Zhao Q Li X-Y Zhang XX. Prevalence of Haemophilus parasuis “Glaesserella parasuis” in pigs in China: a systematic review and meta-analysis. Prev Vet Med. (2020) 182:105083. 10.1016/j.prevetmed.2020.105083 32652336

[25] Dünnwald T Gatterer H Faulhaber M Arvandi M Schobersberger W. Body composition and body weight changes at different altitude levels: a systematic review and meta-analysis. Front Physiol. (2019) 10:430. 10.3389/fphys.2019.00430 31057421 PMC6477059

[26] Barendregt JJ Doi SA Lee YY Norman RE Vos T. Meta-analysis of prevalence. J Epidemiol Community Health. (2013) 67:974–8. 10.1136/jech-2013-203104 23963506

[27] Gong QL Dong L Diao N-C Liu Y Li B-Y Tian T. Mink aleutian disease seroprevalence in China during 1981-2017: a systematic review and meta-analysis. Microb Pathog. (2020) 139:103908. 10.1016/j.micpath.2019.103908 31830583

[28] Wang ZD Wang SC Liu HH Ma HY Li Z-Y Wei F. Prevalence and burden of toxoplasma gondii infection in HIV-infected people: a systematic review and meta-analysis. Lancet HIV. (2017) 4:e177–e188. 10.1016/S2352-3018(17)30005-X 28159548

[29] Ding H Gao YM Deng Y Lamberton PH Lu DB. A systematic review and meta-analysis of the seroprevalence of Toxoplasma gondii in cats in mainland China. Parasit Vectors. (2017) 10:27. 10.1186/s13071-017-1970-6 28086987 PMC5237326

[30] Gong QL Ge GY Wang Q Tian T Liu F Diao NC. Meta-analysis of the prevalence of Echinococcus in dogs in China from 2010 to 2019. PLoS Negl Trop Dis. (2021) 15:e0009268. 10.1371/journal.pntd.0009268 33798191 PMC8018629

[31] Hyera JMK Liess B Frey HR. Bovine viral diarrhoea virus infection in cattle, sheep and goats in Northern Tanzania. Trop Anim Health Prod. (1991) 23:83–94. 10.1007/BF02361187 1650046

[32] Depner K Hübschle OJ Liess B. Prevalence of ruminant pestivirus infections in Namibia. Onderstepoort J Vet Res. (1991) 58:107–9. 10.1080/00480169.1991.35666 1652725

[33] Feknous N Hanon JB Tignon M Khaled H Bouyoucef A Cay B. Seroprevalence of border disease virus and other pestiviruses in sheep in Algeria and associated risk factors. BMC Vet Res. (2018) 14:339. 10.1186/s12917-018-1666-y 30419908 PMC6233519

[34] Lysholm S Ramabu SS Berg M Wensman JJ. First-time detection of bovine viral diarrhoea virus, BVDV-1, in cattle in Botswana. Onderstepoort J Vet Res. (2019) 86:e1–e7. 10.4102/ojvr.v86i1.1764 31714135 PMC6852425

[35] Rosadio RH Evermann JF DeMartini JCA. preliminary serological survey of viral antibodies in Peruvian sheep. Vet Microbiol. (1984) 10:91–6. 10.1016/0378-1135(84)90059-2 6528458

[36] Julia S Craig MI. Jiménez LS Pinto GB Weber EL. First report of BVDV circulation in sheep in Argentina. Prev Vet Med. (2009) 90:274–7. 10.1016/j.prevetmed.2009.05.015 19501923

[37] Okur-Gumusova S Yazici Z Albayrak H. Pestivirus seroprevalence in sheep populations from inland and coastal zones of Turkey. Revue Méd Vét. (2006) 12:595–8.

[38] Ataseven V Ataseven L Tan T Babuer C Oguzoglu T. Seropositivity of agents causing abortion in local goat breeds in Eastern and South-eastern Anatolia, Turkey. Revue De Med Vet. (2006) 157:545–50. 10.1501/0002144

[39] Mishra N Dubey R Rajukumar K Tosh C Tiwari A Pitale SS. Genetic and antigenic characterization of bovine viral diarrhea virus type 2 isolated from Indian goats (Capra hircus). Vet Microbiol. (2007) 124:340–7. 10.1016/j.vetmic.2007.04.023 17509780

[40] Mishra N Pitale SS Pradhan HK. Genetic analysis and expression of NS3 gene of bovine viral diarrhoea virus 1 from India for detection of antibodies in cattle. J Appl Anim Res. (2008) 33:99–103. 10.1080/09712119.2008.9706906

[41] Yeşilbag K Gungor B. Antibody prevalence against respiratory viruses in sheep and goats in North-Western Turkey. Trop Anim Health Prod. (2009) 41:421–5. 10.1007/s11250-008-9225-3 18756353 PMC7088777

[42] Yeşilbag K Alpay G Karakuzulu H. A serologic survey of viral infections in captive ungulates in Turkish zoos. J Zoo Wildl Med. (2011) 42:44–8. 10.1638/2010-0009.1 22946369

[43] Safarpoor Dehkordi F. Prevalence study of Bovine viral diarrhea virus by evaluation of antigen capture ELISA and RT-PCR assay in Bovine, Ovine, Caprine, Buffalo and Camel aborted fetuses in Iran. AMB Express. (2011) 1:32. 10.1186/2191-0855-1-32 22018096 PMC3223133

[44] Giangaspero M Ibata G Savini G Osawa T Tatami S Takagi E. Epidemiological survey of Border disease virus among sheep from northern districts of Japan. J Vet Med Sci. (2011) 73:1629–33. 10.1292/jvms.11-0072 21778667

[45] Li SB. The epidemiological investigation of bovine viral diarrhea virus (BVDV) and infectious bovine rhinotracheitis virus (IBRV) and the identification of isolated IBRV in Liaoning Province. Chinese Acad Agr Sci. (2012).

[46] Oem JK Lee EY Byun JW. Serological and virological investigation of pestiviruses in Korean black goat. Korean J Vet Serv. (2012) 35. 10.7853/kjvs.2012.35.2.129

[47] Kalaiyarasu S Mishra N Rajukumar K Nema RK Behera SP. Development and Evaluation of a Truncated Recombinant NS3 Antigen-Based Indirect ELISA for Detection of Pestivirus Antibodies in Sheep and Goats. J Immunoassay Immunochem. (2015) 36:312–23. 10.1080/15321819.2014.947433 25118572

[48] Mao L Li WL Yang LL Hao F Zhang WW Jiang JY. Discovery and identification of goat-derived BVDV1 and BVDV3. Proceedings of the 16th Academic Symposium of the Chinese Society of Anim Husbandry and Vet Med. (2015) (In Chinese) Available online at: https://kns.cnki.net/kcms/detail/detail.aspx?dbcode=CPFD&dbname=CPFDLAST2016&filename=ZGXJ201509002197&v=J3HOG6q2v2Qbl%25mmd2FB7zrCJtG6psqi%25mmd2B4oL%25mmd2BGGY5rrvTnRG3pu8ry4SEKp0R6DBv0YEqKu3SQM1lGiw%3d

[49] Mao L Li WL Yang LL Wang JH Cheng SP Wei Y. Primary surveys on molecular epidemiology of bovine viral diarrhea virus 1 infecting goats in Jiangsu province, China. BMC Vet Res. (2016) 12:181. 10.1186/s12917-016-0820-7 27596263 PMC5011786

[50] Chen YZ Bao GC Zhang SX Han M. Epidemiological Investigation of Bovine Viral Diarrhea Virus and Sheep Boundary Virus in Tibetan Sheep in Haibei Area, Qinghai Province. Chin J Vet Drug. (2017) 51:7–11.

[51] Bulut H Sozdutmaz I Pestil Z Abayli H Sait A Cevik A. High Prevalence of Bovine Viral Diarrhea Virus-1 in Sheep Abortion Samples with Pestivirus Infection in Turkey. Pakistan Vet J. (2018) 38:71–5. 10.29261/pakvetj/2018.014 30348908

[52] Deng Y Wang S Liu R Hao G. Genetic Diversity of Bovine Viral Diarrhea Virus Infection in Goats in Southwestern China. J Vet Med. (2018) 8274397. 10.1155/2018/8274397 30581873 PMC6276411

[53] Tamer C Palanci H Bayram E Cakmaker M Ozan E Kadi H. Serological data of bovine herpes virus type-1 and bovine viral diarrhea virus infections in various ruminants in small-scale farms in the Central and Eastern Black Sea Region, Turkey. Indian J Anim Res. (2018) 52:903–6. 10.18805/ijar.v0iOF.8470

[54] Ma JG Tian AL Zheng WB Zou Y Zhang YT Yang ZQ. First report of bovine viral diarrhea virus and Mycobacterium avium subspecies paratuberculosis infection in Tibetan sheep (Ovis aries) in Tibetan Plateau, China. Trop Anim Health Prot. (2019) 51:719–22. 10.1007/s11250-018-1718-0 30269235

[55] Hidayat W Wuryastuty H Wasito R. Detection of Pestivirus in small ruminants in Central Java, Indonesia. Vet World. (2021) 14:996–1001. 10.14202/vetworld.2021.996-1001 34083951 PMC8167512

[56] Løken T Krogsrud J Larsen IL. Pestivirus infections in Norway. Serological investigations in cattle, sheep and pigs. Acta Vet Scand. (1991) 32:27–34. 10.1186/BF03546994 1659160 PMC8127921

[57] Løken T. Pestivirus infections in Norway. Epidemiological studies in goats. J Comp Pathol. (1990) 103:1–10. 10.1016/s0021-9975(08)80130-2 2168445

[58] Graham DA Calvert V German A McCullough SJ. Pestiviral infections in sheep and pigs in Northern Ireland. Vet Rec. (2001) 148:69–72. 10.1136/vr.148.3.69 12503593

[59] O'Neill RG O'Connor M O'Reilly PJA. survey of antibodies to pestivirus in sheep in the Republic of Ireland. Ir Vet J. (2004) 57:525–30. 10.1186/2046-0481-57-9-525 21851662 PMC3113815

[60] Aduriz G Atxaerandio R Cortabarria N. First detection of bovine viral diarrhoea virus type 2 in cattle in Spain. Vet Rec Open. (2015) 2:e000110. 10.1136/vetreco-2014-000110 26392905 PMC4567162

[61] Danuser R Vogt HR Kaufmann T Peterhans E Zanoni R. Seroprevalence and characterization of pestivirus infections in small ruminants and new world camelids in Switzerland. Schweiz Arch Tierheilkd. (2009) 151:109–17. 10.1024/0036-7281.151.3.109 19263380

[62] Krametter-Froetscher R Duenser M Preyler B Theiner A Benetka V Moestl K. Pestivirus infection in sheep and goats in West Austria. Vet J. (2010) 186:342–6. 10.1016/j.tvjl.2009.09.006 20042353

[63] Czopowicz M Kaba J Schirrmeier H Bagnicka E Szaluś-Jordanow O Nowicki M. Serological evidence for BVDV-1 infection in goats in Poland - short communication. Acta Vet Hung. (2011) 59:399–404. 10.1556/AVet.2011.022 21727071

[64] Casaubon J Vogt HR Stalder H Hug C Ryser-Degiorgis MP. Bovine viral diarrhea virus in free-ranging wild ruminants in Switzerland: low prevalence of infection despite regular interactions with domestic livestock. BMC Vet Res. (2012) 8:204. 10.1186/1746-6148-8-204 23107231 PMC3514304

[65] Decaro N Lucente MS Lanave G Gargano P Larocca V Losurdo M. Evidence for circulation of bovine viral diarrhoea virus type 2c in ruminants in Southern Italy. Transbound Emerg Dis. (2017) 64:1935–44. 10.1111/tbed.12592 27878974

[66] Emma C James M Joe C Aoibheann D Asa M Andrew WB. Pestivirus apparent prevalence in sheep and goats in Northern Ireland: a serological survey. Vet Rec. (2019) 188:e1. 10.1002/vetr.1 34651766

[67] Potârniche AV Czopowicz M Szalu?-Jordanow O Moroz A Mickiewicz M Witkowski L. Herd-level seroprevalence of pestivirus infection in goat population in Poland. Pol J Vet Sci. (2020) 23:229–33. 10.24425/pjvs.2020.133637 32627993

[68] Robinson AJ. Serological evidence of bovine virus diarrhoea virus is cattle and sheep in the south island of New Zealand. N Z Vet J. (1971) 19:223–4. 10.1080/00480169.1971.33972 5286932

[69] Evans CA Lanyon SR O'Handley RM Reichel MP Cockcroft PD. Seroprevalence of antibodies to Pestivirus infections in South Australian sheep flocks. Aust Vet J. (2018) 96:312–4. 10.1111/avj.12709 30129028

[70] Evans CA Han JH Weston JF Heuer C Gates MC. Serological evidence for exposure to bovine viral diarrhoea virus in sheep co-grazed with beef cattle in New Zealand. N Z Vet J. (2020) 68:238–41. 10.1080/00480169.2019.1705932 31852409

[71] Lamontagne L Roy R. Presence of antibodies to bovine viral diarrhea-mucosal disease virus (border disease) in sheep and goat flocks in Quebec. Can J Comp Med. (1984) 48:225–7. 10.1016/0007-1935(84)90003-4 6326984 PMC1236044

[72] Wang X Yang Q Zhang S Zhang X Pan C Chen H. Genetic Effects of Single Nucleotide Polymorphisms in the Goat GDF9 Gene on Prolificacy: True or False Positive? Animal. (2019) 9:886. 10.3390/ani9110886 31683597 PMC6912770

[73] Deniskova TE Dotsev AV Selionova MI Kunz E Medugorac I Reyer H. Population structure and genetic diversity of 25 Russian sheep breeds based on whole-genome genotyping. Genet Sel Evol. (2018) 50:29. 10.1186/s12711-018-0399-5 29793424 PMC5968526

[74] Wolff PL Schroeder C. McAdoo C Cox M Nelson DD Evermann JF. Evidence of bovine viral diarrhea virus infection in three species of sympatric wild ungulates in nevada: life history strategies may maintain endemic infections in wild populations. Front Microbiol. (2016) 7:292. 10.3389/fmicb.2016.00292 27014215 PMC4783583

[75] Mozaffari AA Khalili M Jahangosha F. Identification of cattle persistently infected with bvdv (pi) by ear-notch testing in southeast of Iran. Anim Rev. (2014) 1:65–8.

[76] Broaddus C Holyoak GR Dawson L Step DL Funk RA Kapil S. Transmission of bovine viral diarrhea virus to adult goats from persistentþy infected cattle. J Vet Diag Invest. (2007) 19:545–7. 10.1177/104063870701900514 17823400

[77] Bauermann FV Ridpath JF. Epidemiology of pestivirus H in Brazil and its control implications. Front Vet Sci. (2021) 8:693041. 10.3389/fvets.2021.693041 34368280 PMC8342886

[78] Hilbe M Stalder H Peterhans E Haessig M Nussbaumer M Egli C. Comparison of five diagnostic methods for detecting bovine viral diarrhea virus infection in calves. J Vet Diagn Invest. (2007) 19:28–34. 10.1177/104063870701900105 17459829

[79] Horner GW Tham KM Orr D Ralston J Rowe S Houghton T. Comparison of an antigen capture enzyme-linked assay with reverse transcription polymerase chain reaction and cell culture immunoperoxidase tests for the diagnosis of ruminant pestivirus infections. Vet Microbiol. (1995) 43:75–84. 10.1016/0378-1135(94)00080-G 7536370

[80] Van Campen H. Epidemiology and control of BVD in the U.S. Vet Microbiol. (2010) 142:94–98. 10.1016/j.vetmic.2009.09.049 19833455

[81] Scharnböck B Roch FF Richter V Funke C Firth CL Obritzhauser W. A meta-analysis of bovine viral diarrhoea virus (BVDV) prevalences in the global cattle population. Sci Rep. (2018) 8:14420. 10.1038/s41598-018-32831-2 30258185 PMC6158279

[82] Rossmanith W Deinhofer M Janacek R Trampler R Wilhelm E. Voluntary and compulsory eradication of bovine viral diarrhoea virus in Lower Austria. Vet Microbiol. (2010) 142:143–9. 10.1016/j.vetmic.2009.09.055 19931989

[83] Presi P Heim D. BVD eradication in Switzerland–a new approach. Vet Microbiol. (2010) 142:137–142. 10.1016/j.vetmic.2009.09.054 19883982

[84] Santman-Berends I Mars MH Van Duijn L Van den Broek KWH Van Schaik G. A quantitative risk-analysis for introduction of Bovine viral diarrhoea virus in the Netherlands through cattle imports. Prev Vet Med. (2017) 146:103–13. 10.1016/j.prevetmed.2017.08.003 28992914

[85] Polak MP Kuta A Rybałtowski W Rola J Larska M Zmudziński JF. First report of bovine viral diarrhoea virus-2 infection in cattle in Poland. Vet J. (2014) 202:643–5. 10.1016/j.tvjl.2014.09.026 25457262

[86] Krametter-Froetscher R Loitsch A Kohler H Schleiner A Schiefer P Moestl K. Prevalence of antibodies to pestiviruses in goats in Austria. J Vet Med B Infect Dis Vet Public Health. (2006) 53:48–50. 10.1111/j.1439-0450.2006.00906.x 16460357

[87] Krametter-Frötscher R Loitsch A Kohler H Schleiner A Schiefer P Möstl K. Serological survey for antibodies against pestiviruses in sheep in Austria. Vet Rec. (2007) 160:726–30. 10.1136/vr.160.21.726 17526894

[88] Mishra N Pitale SS Rajukumar K Prakash A Behera SP Nema RK. Genetic variety of bovine viral diarrhea virus 1 strains isolated from sheep and goats in India. Acta Virol. (2012) 56:209–15. 10.4149/av_2012_03_209 23043600

[89] Houe H. Economic impact of BVDV infection in dairies. Biologicals. (2003) 31:137–43. 10.1016/S1045-1056(03)00030-7 12770546

[90] Spetter MJ Uriarte ELL Altamiranda EAG Leunda MR Pereyra SB Verna AE. Dual natural infection with bovine viral diarrhea virus−1 and−2 in a stillborn calf: tissue distribution and molecular characterization. Open Vet J. (2018) 8:493–7. 10.4314/ovj.v8i4.23 30775291 PMC6356097

[91] Richter V Lebl K Baumgartner W Obritzhauser W Käsbohrer A Pinior B. A systematic worldwide review of the direct monetary losses in cattle due to bovine viral diarrhoea virus infection. Vet J. (2017) 220:80–7. 10.1016/j.tvjl.2017.01.005 28190502

[92] Mishra N Rajukumar K Tiwari A Nema RK Behera SP Satav JS. Prevalence of Bovine viral diarrhoea virus (BVDV) antibodies among sheep and goats in India. Trop Anim Health Prod. (2009) 41:1231–9. 10.1007/s11250-009-9305-z 19153817

[93] Saa L Perea A Garcia-Bocanegra I Arenas A Jara D Ramos P. ‘Seroprevalence and risk factors associated with bovine viral diarrhea virus (BVDV) infection in non-vaccinated dairy and dual purpose cattle herds in Ecuador’. Tropical Animal Health and Production. (2012) 44:645–9. 10.1007/s11250-011-9948-4 21822791

