# Supplementary material for: Prevalence of Bovine Viral Diarrhea Virus in Ovine and Caprine Flocks: A Global Systematic Review and Meta-Analysis
Source: Front Vet Sci. 2021 Nov 19;8:703105. doi: 10.3389/fvets.2021.703105 (PMC8639873; doi:10.3389/fvets.2021.703105)

Supplementary Material 1. Items on the PRISMA checklist.

Section/topic	#	Checklist item	Reported on page #
**TITLE**			
Title	1	Prevalence of bovine viral diarrhea virus (BVDV) in ovine and caprine flocks: a global systematic review and meta-analysis	1
**ABSTRACT**			
Structured summary	2	Provide a structured summary including, as applicable: background; objectives; data sources; study eligibility criteria, participants, and interventions; study appraisal and synthesis methods; results; limitations; conclusions and implications of key findings; systematic review registration number.	1-2
**INTRODUCTION**			
Rationale	3	Describe the rationale for the review in the context of what is already known.	3
Objectives	4	Provide an explicit statement of questions being addressed with reference to participants, interventions, comparisons, outcomes, and study design (PICOS).	3
**METHODS**			
Protocol and registration	5	Indicate if a review protocol exists, if and where it can be accessed (e.g., Web address), and, if available, provide registration information including registration number.	4
Eligibility criteria	6	Specify study characteristics (e.g., PICOS, length of follow-up) and report characteristics (e.g., years considered, language, publication status) used as criteria for eligibility, giving rationale.	4
Information sources	7	Describe all information sources (e.g., databases with dates of coverage, contact with study authors to identify additional studies) in the search and date last searched.	4
Search	8	Present full electronic search strategy for at least one database, including any limits used, such that it could be repeated.	4
Study selection	9	State the process for selecting studies (i.e., screening, eligibility, included in systematic review, and, if applicable, included in the meta-analysis).	4-5

Data collection process	10	Describe method of data extraction from reports (e.g., piloted forms, independently, in duplicate) and any processes for obtaining and confirming data from investigators.	5
Data items	11	List and define all variables for which data were sought (e.g., PICOS, funding sources) and any assumptions and simplifications made.	5
Risk of bias in individual studies	12	Describe methods used for assessing risk of bias of individual studies (including specification of whether this was done at the study or outcome level), and how this information is to be used in any data synthesis.	5-6
Summary measures	13	State the principal summary measures (e.g., risk ratio, difference in means).	5-6
Synthesis of results	14	Describe the methods of handling data and combining results of studies, if done, including measures of consistency (e.g., I^2) for each meta-analysis.	5-6
Risk of bias across studies	15	Specify any assessment of risk of bias that may affect the cumulative evidence (e.g., publication bias, selective reporting within studies).	5-6
Additional analyses	16	Describe methods of additional analyses (e.g., sensitivity or subgroup analyses, meta-regression), if done, indicating which were pre-specified.	5-6
**RESULTS**			
Study selection	17	Give numbers of studies screened, assessed for eligibility, and included in the review, with reasons for exclusions at each stage, ideally with a flow diagram.	6-7, Figure 1, Tables 1
Study characteristics	18	For each study, present characteristics for which data were extracted (e.g., study size, PICOS, follow-up period) and provide the citations.	6-7, Tables 2 and 3
Risk of bias within studies	19	Present data on risk of bias of each study and, if available, any outcome level assessment.	7, Figures 2 and 3, Figures S1-S2, Table S1and S2
Results of individual studies	20	For all outcomes considered (benefits or harms), present, for each study: (a) simple summary data for each intervention group (b) effect estimates and confidence intervals, ideally with a forest plot.	7, Figure 4 and 5
Synthesis of results	21	Present results of each meta-analysis done, including confidence intervals and measures of consistency.	7, Tables 4 and 5, Table 6 and 7

Risk of bias across studies	22	Present results of any assessment of risk of bias across studies.	7, Figures S3 and S4, Table S1 and S2
Additional analysis	23	Give results of additional analyses, if done (e.g., sensitivity or subgroup analyses, meta-regression.)	7-8, Figure S5 and S6
**DISCUSSION**			
Summary of evidence	24	Summarize the main findings including the strength of evidence for each main outcome; consider their relevance to key groups (e.g., healthcare providers, users, and policy makers).	8-9
Limitations	25	Discuss limitations at study and outcome level (e.g., risk of bias), and at review-level (e.g., incomplete retrieval of identified research, reporting bias).	8-9
Conclusions	26	Provide a general interpretation of the results in the context of other evidence, and implications for future research.	10
**FUNDING**			
Funding	27	Describe sources of funding for the systematic review and other support (e.g., supply of data); role of founders for the systematic review.	11

From: Moher D, Liberati A, Tetzlaff J, Altman DG, The PRISMA Group (2009). Preferred Reporting Items for Systematic Reviews and Meta-Analyses: The PRISMA Statement. PLoS Med 6(6): e1000097. doi:10.1371/journal.pmed1000097

For more information, visit: **www.prisma-statement.org**.

Supplementary Material 2. Search strategies and restrictions.

Database	Limitation	Search formula*
PubMed*	All files	(Diarrhea Viruses, Bovine Viral [Mesh] OR BVDV OR Diarrhea Virus, Bovine Viral OR Bovine Viral Diarrhea Viruses OR Bovine Diarrhea Virus OR Bovine Diarrhea Viruses OR Diarrhea Virus, Bovine OR Diarrhea Viruses, Bovine OR Virus, Bovine Diarrhea OR Viruses, Bovine Diarrhea OR Bovine Pestivirus OR Bovine Pestiviruses OR Pestiviruses, Bovine) AND (sheep [MeSH Terms] OR Ovis OR Dall Sheep OR Ovis dalli OR Sheep, Dall OR Goats [MeSH Terms] OR Goat OR Capra OR Capras OR Sheep, Domestic OR Domestic Sheep OR Ovis ammon aries OR Ovis aries OR Mouflon OR Mouflons OR Ovis gmelini musimon OR Ovis aries musimon OR Small ruminants [MeSH Terms])
ScienceDirect	All files	(“Diarrhea Viruses, Bovine Viral” OR BVDV OR Pestivirus) AND (sheep OR goat OR small ruminants) AND (prevalence) within Research articles
CNKI	Advanced Search & Subject term & Fuzzy retrieval and synonym extension	“BVDV” + “Viral diarrhea” AND “sheep and goats” + “small ruminants” (The words were spelled in Chinese; the “+” means “OR”, the Boolean operator)
Chongqing VIP	Advanced Search & Title or keyword & Fuzzy retrieval and synonym extension	(BVDV OR Viral diarrhea) AND (sheep and goats OR small ruminants) (The words were spelled in Chinese and “OR”)

WanFang	Papers in journals, degree theses, and conferences. Advanced Search & Title or keyword & Fuzzy retrieval and synonym extension	(BVDV OR Viral diarrhea) AND (sheep and goats OR small ruminants) (in Chinese)
Springer-Link	All files	("Diarrhea Viruses, Bovine Viral" OR BVDV OR Pestivirus) AND (sheep OR goat OR small ruminants) AND (prevalence) within Article.
Web of Science	Keywords & Searched for the TOPIC	("Diarrhea Viruses, Bovine Viral" OR BVDV OR Pestivirus) AND (sheep OR goat OR small ruminants) AND (prevalence) within Articles.

* The different search strategy was used because different databases have different retrieval logic.

Supplementary Material 3. Code.

PRAW	Rate < -transform(m1, r = event/n)<br>shapiro.test(rate$r)
PLN	Rate < -transform(m1, log = log(event/n))<br>shapiro.test(rate$log)
PLOGIT	Rate < -transform(m1, logit = log((event/n)/(1-event/n)))<br>shapiro.test(rate$logit)
PAS	Rate < -transform(m1, arcsin.size = asin(sqrt(event/(n+1))))<br>shapiro.test(rate$arcsin)
PFT	Rate < -transform(m1,darcsin =<br>0.5*(asin(sqrt(event/(n+1)))+asin((sqrt(event+1)/(n+1)))))<br>shapiro.test(rate$darcsin)

Supplementary Material 4. Included studies and their quality scores.

Study No.	Reference ID	No. tested	No. positive	Prevalence	Random sampling or not	Detection method clearly or not	Sample size greater than or equal to 200	Sampled time clearly or not	Four or more risk factors or not	Score	Study Quality
1	Robinson (1971)	50	14	28.00%	N	Y	N	N	N	1	Low
2	Rosadio et al. (1984)	34	1	2.94%	N	Y	N	N	Y	2	middle
3	Lamontagne et al.(1984)	799	89	11.14%	N	Y	Y	N	Y	3	middle
4	Løken. (1990)	2335	83	3.55%	N	Y	Y	Y	Y	4	high
5	Hyera et al. (1991)	1564	424	27.11%	Y	Y	Y	Y	Y	5	high
6	Depner et al. (1991)	1736	137	78.91%	Y	Y	Y	Y	Y	5	high
7	Løken et al. (1991)	3712	166	4.47%	N	Y	Y	Y	Y	4	high

8	Graham et al. (2001)	918	14	1.53%	N	Y	Y	Y	Y	4	high
9	O'Neill et al. (2004)	1448	39	2.69%	N	Y	Y	Y	Y	4	high
10	Okur-Gumusova et al. (2006)	2444	463	18.94%	Y	Y	Y	N	Y	4	high
11	Ataseven et al. (2006)	275	83	30.18%	Y	Y	Y	Y	Y	5	high
12	Krametter-Froetscher et al. (2006)	549	32	5.83%	N	Y	Y	N	Y	3	middle
13	Mishra et al. (2007)	562	2	0.36%	Y	Y	Y	Y	Y	5	high
14	Mishra et al. (2008)	1561	8	0.51%	Y	Y	Y	Y	Y	5	high
15	Yeşilbağ and Gungor (2009)	388	124	31.96%	Y	Y	Y	Y	Y	5	high
16	Danuser et al. (2009)	5562	827	14.87%	Y	Y	Y	Y	Y	5	high
17	Julia' et al. (2009)	54	43	79.63%	N	Y	N	N	Y	2	middle
18	Krametter-Froetsche et al. (2010)	1196	1	0.08%	N	Y	Y	Y	Y	4	high
19	Yeşilbağ et al. (2011)	137	0	0.00%	N	Y	N	N	Y	2	middle
20	Safarpoor Dehkordi (2011)	967	167	35.14%	N	Y	Y	Y	Y	4	high
21	Giangaspero et al. (2011)	165	1	0.61%	N	Y	N	Y	Y	3	middle
22	Czopowicz et al. (2011)	1060	7	0.66%	Y	Y	Y	Y	Y	5	high
23	Li (2012)	202	92	45.54%	N	Y	Y	N	Y	3	middle
24	Casaubon et al. (2012)	500	9	1.80%	N	Y	Y	Y	Y	4	high
25	Oem et al. (2012)	672	10	1.49%	Y	Y	Y	Y	Y	5	high
26	Mao et al. (2015)	238	31	13.03%	N	Y	Y	Y	Y	4	high
27	Kalaiyarasu et al. (2015)	569	187	32.86%	N	Y	Y	Y	Y	4	high
28	Mao et al. (2016)	236	29	12.29%	N	Y	Y	Y	Y	4	high
29	Chen et al. (2017)	195	58	29.74%	N	Y	N	Y	Y	3	middle
30	Decaro et al. (2017)	1231	27	2.19%	N	Y	Y	Y	Y	4	high
31	Tamer et al. (2018)	543	101	18.60%	Y	Y	Y	N	Y	4	high
32	Deng et al. (2018)	217	38	17.51%	N	Y	Y	Y	Y	4	high
33	Evans et al. (2018)	875	0	0.00%	Y	Y	Y	N	Y	4	high

34	Feknous et al. (2018)	689	2	0.29%	N	Y	Y	N	Y	3	middle
35	Bulut et al. (2018)	396	40	10.10%	N	Y	Y	Y	Y	4	high
36	Silveira et al. (2018)	500	20	4.00%	N	Y	Y	Y	Y	4	high
37	Lysholm et al. (2019)	100	0	0.00%	Y	Y	N	Y	Y	4	high
38	Ma et al. (2019)	2187	804	36.76%	Y	Y	Y	Y	Y	5	high
39	Emma et al. (2019)	3372	56	1.66%	Y	Y	Y	Y	Y	5	high
40	Potârniche et al. (2020)	910	7	0.77%	Y	Y	Y	Y	Y	5	high
41	Evans et al. (2020)	270	17	6.30%	N	Y	Y	Y	Y	4	high
42	Hidayat et al. (2021)	46	2	4.35%	Y	Y	N	Y	Y	4	high

[a]N: No;
[b]Y: Yes.

1. Robinson AJ. Serological evidence of bovine virus diarrhoea virus is cattle and sheep in the south island of New Zealand. *N Z Vet J*. (1971) 19:223-4. doi: 10.1080/00480169.1971.33972
2. Rosadio RH, Evermann JF, DeMartini JC. A preliminary serological survey of viral antibodies in Peruvian sheep. *Vet Microbiol*. (1984) 10:91-6. doi: 10.1016/0378-1135(84)90059-2
3. Lamontagne L, Roy R. Presence of antibodies to bovine viral diarrhea-mucosal disease virus (border disease) in sheep and goat flocks in Quebec. *Can J Comp Med*. (1984) 48:225-7. doi: 10.1016/0007-1935(84)90003-4
4. Løken T. Pestivirus infections in Norway. Epidemiological studies in goats. *J Comp Pathol*. (1990) 103:1-10. doi: 10.1016/s0021-9975(08)80130-2
5. Hyera JMK, Liess B, Frey HR. Bovine Viral Diarrhoea Virus Infection in Cattle, Sheep and Goats in Northern Tanzania. *Trop Anim Health Prod*. (1991) 23:83-94. doi: 10.1007/BF02361187
6. Depner K, Hübschle OJ, Liess B. Prevalence of ruminant pestivirus infections in Namibia. *Onderstepoort J Vet Res*. (1991) 58:107-9. doi:10.1080/00480169.1991.35666
7. Løken T, Krogsrud J, Larsen IL. Pestivirus infections in Norway. Serological investigations in cattle, sheep and pigs. *Acta Vet Scand*. (1991) 32:27-34. doi: 10.1186/BF03546994

8. Graham DA, Calvert V, German A, et al. Pestiviral infections in sheep and pigs in Northern Ireland. *Vet Rec*. (2001) 148:69-72. doi: 10.1136/vr.148.3.69
9. O'Neill RG, O'Connor M, O'Reilly PJ. A survey of antibodies to pestivirus in sheep in the Republic of Ireland. *Ir Vet J*. (2004) 57:525-30. doi: 10.1186/2046-0481-57-9-525
10. Okur-Gumusova S ,Yazici Z, Albayrak H. Pestivirus seroprevalence in sheep populations from inland and coastal zones of Turkey. *Revue Méd Vét*. (2006) 12:595-8.
11. Ataseven V, Ataseven L, Tan T, Babuer C, Oguzoglu T, et al. Seropositivity of agents causing abortion in local goat breeds in Eastern and South-eastern Anatolia, Turkey. *Revue De Med Vet*. (2006) 157:545-50. doi: 10.1501/0002144
12. Krametter-Froetscher R, Loitsch A, Kohler H, et al. Prevalence of antibodies to pestiviruses in goats in Austria. *J Vet Med B Infect Dis Vet Public Health*. (2006) 53:48-50. doi: 10.1111/j.1439-0450.2006.00906.x
13. Mishra N, Dubey R, Rajukumar K, Tosh C, Tiwari A, Pitale SS, et al. Genetic and antigenic characterization of bovine viral diarrhea virus type 2 isolated from Indian goats (Capra hircus). *Vet Microbiology*. (2007) 124:340-7. doi: 10.1016/j.vetmic.2007.04.023
14. Mishra N, Rajukumar K, Vilcek S, Tiwari A, Satav JS, Dubey SC. Molecular characterization of bovine viral diarrhea virus type 2 isolate originating from a native Indian sheep (Ovies aries). *Vet Microbiology*. (2008) 130:88-98. doi: 10.1016/j.vetmic.2008.01.005
15. Yesilbag K, Gungor B. Antibody prevalence against respiratory viruses in sheep and goats in North-Western Turkey. *Trop Anim Health Prod*. (2009) 41:421-5. doi: 10.1007/s11250-008-9225-3
16. Danuser R, Vogt HR, Kaufmann T, Peterhans E, Zanoni R. Seroprevalence and characterization of pestivirus infections in small ruminants and new world camelids in Switzerland. *Schweiz Arch Tierheilkd*. (2009) 151:109-17. doi: 10.1024/0036-7281.151.3.109
17. Julia S, Craig MI, Jime´nez LS, Pinto GB, Weber EL. First report of BVDV circulation in sheep in Argentina. *Prev Vet Med*. (2009) 90:274-7. doi: 10.1016/j.prevetmed.2009.05.015
18. Krametter-Froetscher R, Duenser M, Preyler B, Theiner A, Benetka V, Moestl K, et al. Pestivirus infection in sheep and goats in West Austria. *Vet J*. (2010) 186:342-6. doi: 10.1016/j.tvjl.2009.09.006
19. Yesilbag K, Alpay G, Karakuzulu H. A serologic survey of viral infections in captive ungulates in Turkish zoos. *J Zoo Wildl Med*. (2011) 42:44-8. doi: 10.1638/2010-0009.1
20. Safarpoor Dehkordi F. Prevalence study of Bovine viral diarrhea virus by evaluation of antigen capture ELISA and RT-PCR assay in Bovine, Ovine, Caprine, Buffalo and Camel aborted fetuses in Iran. *AMB Express*. (2011) 1:32. doi: 10.1186/2191-0855-1-32
21. Giangaspero M, Ibata G, Savini G, Osawa T, Tatami S, Takagi E, et al. Epidemiological survey of Border disease virus among sheep from northern districts of Japan. *J Vet Med Sci*. (2011) 73:1629-33. doi: 10.1292/jvms.11-0072

22. Czopowicz M, Kaba J, Schirrmeier H, Bagnicka E, Szaluś-Jordanow O, Nowicki M, et al. Serological evidence for BVDV-1 infection in goats in Poland - short communication. *Acta Vet Hung*. (2011) 59:399-404. doi: 10.1556/AVet.2011.022
23. Li SB. The epidemiological investigation of bovine viral diarrhea virus (BVDV) and infectious bovine rhinotracheitis virus (IBRV) and the identification of isolated IBRV in Liaoning Province. *Chinese Acad Agr Sci*. (2012) (In Chinese) https://kns.cnki.net/kcms/detail/detail.aspx?dbcode=CMFD&dbname=CMFD201302&filename=1013173375.nh&v=qxHQrl3cYsN%25mmd2BIt7UX%25mmd2Fag3TlxBk9NyBx2BCRrvD9ixBPUwp%25mmd2FHUgI9JZ%25mmd2FVG7cYqxhd
24. Casaubon J, Vogt HR, Stalder H, Hug C, Ryser-Degiorgis MP. Bovine viral diarrhea virus in free-ranging wild ruminants in Switzerland: low prevalence of infection despite regular interactions with domestic livestock. *BMC Vet Res*. (2012) 8:204. doi: 10.1186/1746-6148-8-204
25. Oem JK, Lee EY, Byun JW, et al. Serological and virological investigation of pestiviruses in Korean black goat. *Korean J Vet Serv*.(2012) 35. doi: 10.7853/kjvs.2012.35.2.129
26. Mao L, Li WL, Yang LL, Hao F, Zhang WW, Jiang JY. Discovery and identification of goat-derived BVDV1 and BVDV3. *Proceedings of the 16th Academic Symposium of the Chinese Society of Anim Husbandry and Vet Med*. (2015) (In Chinese) https://kns.cnki.net/kcms/detail/detail.aspx?dbcode=CPFD&dbname=CPFDLAST2016&filename=ZGXJ201509002197&v=J3HOG6q2v2Qbl%25mmd2FB7zrCJtG6psqi%25mmd2B4oL%25mmd2BGGY5rrvTnRG3pu8ry4SEKp0R6DBv0YEqKu3SQM1lGiw%3d
27. Kalaiyarasu S, Mishra N, Rajukumar K, Nema RK, Behera SP. Development and Evaluation of a Truncated Recombinant NS3 Antigen-Based Indirect ELISA for Detection of Pestivirus Antibodies in Sheep and Goats. *J Immunoassay Immunochem*. (2015) 36:312-23. doi: 10.1080/15321819.2014.947433
28. Mao L, Li WL, Yang LL, Wang JH, Cheng SP, Wei Y, et al. Primary surveys on molecular epidemiology of bovine viral diarrhea virus 1 infecting goats in Jiangsu province, China. *BMC Vet Res*. (2016) 12:181. doi: 10.1186/s12917-016-0820-7
29. Chen YZ, Bao GC, Zhang SX, Han M. Epidemiological Investigation of Bovine Viral Diarrhea Virus and Sheep Boundary Virus in Tibetan Sheep in Haibei Area, Qinghai Province. *CHIN J Vet Drug*. (2017) 51:7-11. (In Chinese) https://kns.cnki.net/kcms/detail/detail.aspx?dbcode=CJFD&dbname=CJFDLAST2017&filename=ZSYY201709002&v=PdMirwM2zxWjGZwJNxDtxuBvG%25mmd2FQRTOZEj%25mmd2Bl4Nrh1jdz7iBHPDNixrqMeQWRohPds
30. Decaro N, Lucente MS, Lanave G, Gargano P, Larocca V, Losurdo M, et al. Evidence for Circulation of Bovine Viral Diarrhoea Virus Type 2c in Ruminants in Southern Italy. *Transbound Emerg Dis*. (2017) 64:1935-44. doi: 10.1111/tbed.12592
31. Tamer C, Palanci H, Bayram E, et al. Serological data of bovine herpes virus type-1 and bovine viral diarrhea virus infections in various ruminants in small-scale farms in the Central and Eastern Black Sea Region, Turkey. *Indian J Anim Res*. (2018) 52:903-6. doi: 10.18805/ijar.v0iOF.8470

32. Deng Y, Wang S, Liu R, Hao G. Genetic Diversity of Bovine Viral Diarrhea Virus Infection in Goats in Southwestern China. *J Vet Med*. (2018):8274397. doi: 10.1155/2018/8274397
33. Evans CA, Lanyon SR, O'Handley RM, Reichel MP, Cockcroft PD. Seroprevalence of antibodies to Pestivirus infections in South Australian sheep flocks. *Aust Vet J*. (2018) 96:312-4. doi: 10.1111/avj.12709
34. Feknous N, Hanon JB, Tignon M, Khaled H, Bouyoucef A, Cay B. Seroprevalence of border disease virus and other pestiviruses in sheep in Algeria and associated risk factors. *BMC Vet Res*. (2018) 14:339. doi: 10.1186/s12917-018-1666-y
35. Bulut H, Sozdutmaz I, Pestil Z, Abayli H, Sait A, Cevik A, et al. High Prevalence of Bovine Viral Diarrhea Virus-1 in Sheep Abortion Samples with Pestivirus Infection in Turkey. *Pakistan Vet J*. (2018) 38:71-5. doi: 10.29261/pakvetj/2018.014
36. Silveira S, Falkenberg SM, Elderbrook MJ, Sondgeroth KS, Dassanayake RP, Neill JD, et al. Serological survey for antibodies against pestiviruses in Wyoming domestic sheep. *Vet Microbiol*. (2018) 219:96-9. doi: 10.1016/j.vetmic.2018.04.019
37. Lysholm S, Ramabu SS, Berg M, Wensman JJ. First-time detection of bovine viral diarrhoea virus, BVDV-1, in cattle in Botswana. *Onderstepoort J Vet*. (2019) 86:e1-e7. doi: 10.4102/ojvr.v86i1.1764
38. Ma JG, Tian AL, Zheng WB, Zou Y, Zhang YT, Yang ZQ. First report of bovine viral diarrhea virus and Mycobacterium avium subspecies paratuberculosis infection in Tibetan sheep (Ovis aries) in Tibetan Plateau, China. *Trop Anim Health Pro*. (2019) 51:719-22. doi: 10.1007/s11250-018-1718-0
39. Emma C, James M, Joe C, Aoibheann D, Asa M, Andrew WB, et al. Pestivirus apparent prevalence in sheep and goats in Northern Ireland: A serological survey. *Vet Rec*. (2021) 188:e1. doi: 10.1002/vetr.1
40. Potârniche AV, Czopowicz M, Szaluś-Jordanow O, Moroz A, Mickiewicz M, Witkowski L, et al. Herd-level seroprevalence of pestivirus infection in goat population in Poland. *Pol J Vet Sci*. (2020) 23:229–33. doi: 10.24425/pjvs.2020.133637
41. Evans CA, Han JH, Weston JF, Heuer C, Gates MC. Serological evidence for exposure to bovine viral diarrhoea virus in sheep co-grazed with beef cattle in New Zealand. *N Z Vet J*. (2020) 68:238-41. doi: 10.1080/00480169.2019.1705932
42. Hidayat W, Wuryastuty H, Wasito R. Detection of Pestivirus in small ruminants in Central Java, Indonesia. *Vet World*. (2021) 14:996-1001. doi: 10.14202/vetworld.2021.996-1001

Supplementary Material 5. Excluded articles and causes.

**Reason 1: Summary (1 article)**

1. LOKEN T. Pestivirus infection in ruminants in norway. *Rev Sci Tech*. (1992) 11: 895-9. doi: 10.1088/0004-637X/752/2/125
**Reason 2: Incomplete, unclear, or obviously erroneous data that could not be resolved by contacting the authors (14 articles)**
1. Fernández-Sirera L, Cabezón O, Dematteis A, et al. Survey of Pestivirus infection in wild and domestic ungulates from south-western Italian Alps. *EUR J WILDLIFE RES*. (2012) 58:425-31. doi: 10.1007/s10344-011-0591-1
2. Mishra N, Pitale SS, Rajukumar K, Prakash A, Behera SP, Nema RK, et al. Genetic variety of bovine viral diarrhea virus 1 strains isolated from sheep and goats in India. *Acta Virol*. (2012) 56:209-15. doi: 10.4149/av_2012_03_209
3. Fernandez-Sirera L, Cabezon O, Rossi L, et al. Investigations of pestivirus infection in wild Caprinae in Europe. *Vet Rec*. (2011) 169:15. doi: 10.1136/vr.d1831
4. Tutuncu M, Duz E, Karaca M, Akkan HA, Keles I, Bakir B, Tasal I. A serological investigation of pestiviruses in sheep in eastern border of Turkey. *Trop Anim Health Prod*. (2011) 43:1467-9. doi: 10.1007/s11250-011-9832-2
5. Zaghawa A. Prevalence of antibodies to bovine viral diarrhoea virus and/or border disease virus in domestic ruminants. *Zentralblatt Veterinarmedizin B*. (1998) 45:345-51. doi: 10.1111/j.1439-0450.1998.tb00803.x
6. Yang DK, Kweon CH, Kim BH, et al. Prevalence and genotypes of pestivirus in Korean goats. *Korean J of Vet Res*. (2008) 48:83-8. http://www.koreascience.or.kr/article/JAKO200810103410991.page
7. Marreros N, Hüssy D, Albini S, Frey CF, Abril C, Vogt HR, et al. Epizootiologic investigations of selected abortive agents in free-ranging Alpine ibex (Capra ibex ibex) in Switzerland. *J Wildl Dis*. (2011) 47:530-43. doi: 10.7589/0090-3558-47.3.530
8. Lundén A, Carlsson U, Näslund K. Toxoplasmosis and border disease in 54 Swedish sheep flocks. Seroprevalence and incidence during one gestation period. *Acta Vet Scand*. (1992) 33:175-84. doi: 10.1186/BF03547324

9. Marco I, Rosell R, Cabezón O, Beneria M, Mentaberre G, Casas E, et al. Serologic and virologic investigations into pestivirus infection in wild and domestic ruminants in the Pyrenees (NE Spain). *Res Vet Sci*. (2009) 87:149-53. doi: 10.1016/j.rvsc.2008.10.01
10. Mishra N, Rajukumar K, Tiwari A, Nema RK, Behera SP, Satav, JS, et al. Prevalence of bovine viral diarrhoea virus (bvdv) antibodies among sheep and goats in india. *Trop Anim Health Prod*. (20090 41:1231-9. doi: 10.1007/s11250-009-9305-z
11. Hanon JB, Cay B. Seroprevalence of pestivirus infections is low in Belgian small ruminant flocks and is significantly associated with the presence of cattle. *Prev Vet Med*. (2021) 195:105446. doi: 10.1016/j.prevetmed.2021.105446
12. Lamontagne L, Roy R. Presence of antibodies to bovine viral diarrhea-mucosal disease virus (border disease) in sheep and goat flocks in Quebec. *Can J Comp Med*. (1984) 48:225-7. https://pubmed.ncbi.nlm.nih.gov/6326984/
13. Orsel K, Antonis AF, Oosterloo JC, Vellema P, van der Meer FJ. Seroprevalence of antibodies against pestiviruses in small ruminants in The Netherlands. *TIJDSCHR DIERGENEESK*. (2009) 134:380-4. https://pubmed.ncbi.nlm.nih.gov/19480144/
14. Paniagua J, García-Bocanegra I, Arenas-Montes A, Berriatua E, Espunyes J, Carbonero A, et al. Absence of circulation of Pestivirus between wild and domestic ruminants in southern Spain. *Vet Rec*. (2016) 178:215. doi: 10.1136/vr.103490
**Reason 3: Not a BVDV virus-5 articles**
1. Gómez-Guillamón F, Díaz-Cao JM, Camacho-Sillero L, Cano-Terriza D, Alcaide EM, Cabezón Ó, et al. Spatiotemporal monitoring of selected pathogens in Iberian ibex (Capra pyrenaica). *Transbound Emerg Dis*. (2020). doi: 10.1111/tbed.13576
2. Silveira S, Baumbach LF, Weber MN, Mósena ACS, da Silva MS, Cibulski SP, et al. HoBi-like is the most prevalent ruminant pestivirus in Northeastern Brazil. *Transbound Emerg Dis*. (2018) 65:e113-e120. doi: 10.1111/tbed.12689
3. Sands JJ, Harkness JW. The distribution of antibodies to Border disease virus among sheep in England and Wales. *Res Vet Sci*. (1978) 25:241-2. doi: 10.1016/S0034-5288(18)32987-4

4. Kirkbride CA, Johnson MW. Serologic examination of aborted ovine and bovine fetal fluids for the diagnosis of border disease, bluetongue, bovine viral diarrhea, and leptospiral infections. *J Vet Diagn Invest*. (1989) 1:132-8. doi: 10.1177/104063878900100208
5. HA*E Özan. The Investigation of Pestivirus and Rift Valley Fever Virus Infections in Aborted Ruminant Foetuses in the Blacksea Region in Turkey. *Kafkas niversitesi Veteriner Fakültesi Dergisi*. (2012) 18:457-61. doi: 10.1501/Vetfak_0000002518
**Reason 4: The number of studies is less than 30 (1 articles)**
1. Braun U, Bachofen C, Schenk B, Haessig M, Peterhans E, et al. Investigation of border disease and bovine virus diarrhoea in sheep from 76 mixed cattle and sheep farms in eastern Switzerland. *Schweiz Arch Tierheilkd*. (2013) 155:293-8. doi: 10.1024/0036-7281/a000460
**Reason 5: Research on BVDV vaccination (1 article)**
1. Evans CA, Lanyon SR, Reichel MP. Investigation of AGID and two commercial ELISAs for the detection of Bovine viral diarrhea virus-specific antibodies in sheep serum. *J Vet Diagn Invest*. (2017) 29:181-5. doi: 10.1177/1040638716687003
**Reason 6: Other species besides sheep and goats (1 article)**
1. Mudry M, Meylan M, Regula G, Steiner A, Zanoni R, Zanolari P. Epidemiological study of pestiviruses in South American camelids in Switzerland. *J Vet Intern Med*. (2010) 24:1218-23. doi: 10.1111/j.1939-1676.2010.0577.x
**Reason 7: Non-Chinese and English articles (5 articles)**
1. Daniel FB, Hermelinda RG, Ch CG, et al. Anticuerpos contra el virus de la diarrea viral bovina y su asociación con problemas reproductivos en borregas de una empresa ovejera de la sierra central del Perú. Revista De Investigaciones Veterinarias Del Peru. (2009) 21:113-8. https://www.researchgate.net/publication/262550978_Anticuerpos_contra_el_virus_de_la_diarr

ea_viral_bovina_y_su_asociacion_con_problemas_reproductivos_en_borregas_de_una_empresa_ovejera_de_la_sierra_central_del_Peru
2. Cabello RK, Karina; Quispe ChR, Rivera GH. Frecuencia de los virus Parainfluenza-3, Respiratorio Sincitial y Diarrea Viral Bovina en un rebao mixto de una comunidad campesina de Cusco. *Rev Inv Vet Perú*. (2006) 17: 167-72. https://www.redalyc.org/pdf/3718/371838845014.pdf
3. Silva M, Pituco EM, Lima M, et al. Prevalence of anti-pestivirus antibodies and risk factors in dairy goats from the semiarid region of Paraíba State, Northeastern Brazil. *semina ciências agrárias*. (2014). doi:10.5433/1679-0359.2014v35n3p1291
4. Nathalie LA, Hermelinda RG, Mariluz AR, Néstor FP. Seroprevalencia de pestivirus de rumiantes en ovinos reproductores de una empresa de la sierra central del perú. Rev Investig Vet Per. (2012) 23:504-9. doi:10.15381/rivep.v23i4.971
5. Krametter-Frötscher R, Loitsch A, Kohler H, Schleiner A, Schiefer P, Möstl K, et al. Baumgartner W. Serological survey for 55 antibodies against pestiviruses in sheep in Austria. *Vet Rec*. (2007) 60:726-30. doi: 10.1136/vr.160.21.726
**Reason 8: Cannot download articles (16 articles)**
1. Goyal SM, Khan MA, McPherson SW, Robinson RA, Boylan WJ. Prevalence of antibodies to seven viruses in a flock of ewes in Minnesota. *Am J Vet Res*. (1988) 49:464-7. https://pubmed.ncbi.nlm.nih.gov/2837112/
2. Fulton RW, Downing MM, Hagstad HV. Prevalence of bovine herpesvirus-1, bovine oral diarrhea, parainfluenza-3, bovine adenoviruses-3 and -7, and goat respiratory syncytial viral antibodies in goats. *Am J Vet Res*. (1982) 43:1454-7. https://pubmed.ncbi.nlm.nih.gov/6285781/
3. Elazhary MA, Silim A, Dea S. Prevalence of antibodies to bovine respiratory syncytial virus, bovine viral diarrhea virus, bovine herpesvirus-1, and bovine parainfluenza-3 virus in sheep and goats in Quebec. *Am J Vet Res*. (1984) 45:1660-2. https://pubmed.ncbi.nlm.nih.gov/6089626/
4. Thür B, Caplazi P, Hilbe M, Zlinszky K, Strasser M, Corboz L, et al. [Pestivirus as causative agent of abortion and perinatal mortality in cattle and sheep in Switzerland]. *Dtsch Tierarztl*

Wochenschr. (1998) 105:145-8. https://pubmed.ncbi.nlm.nih.gov/9618985/
5. Schleiner A, Krametter-Fröbtscher R, Schiefer P, Loitsch A, Golja F, Möstl K, et al. [Seroepidemiological survey of sheep in Carinthia for the dissemination of ruminant pestiviruses]. *Berl Munch Tierarztl Wochenschr*. (2006) 119:203-8. https://pubmed.ncbi.nlm.nih.gov/16729466/
6. Niemi SM, Evermann JF, Huffman EM, Kirk JH. Border disease virus isolation from postpartum ewes. *Am J Vet Res*. (1982) 43:86-8. https://pubmed.ncbi.nlm.nih.gov/7103215/
7. Burgu I, Ozturk F, Akzca Y, Toker A, Frey HR, Liess B, et al. Investigations on the occurrence and impact of bovine viral diarrhea (BVD) virus infections in sheep in Turkey. *Dtsch Tierarztl Wochenschr*. (1987) 94:292-4. https://pubmed.ncbi.nlm.nih.gov/3038498/
8. Bohrmann R, Frey H, Liess B, et al. Survey on the prevalence of neutralizing antibodies to bovine viral diarrhea (BVD) virus, bovine herpes virus type 1 (BHV-1) and parainfluenza virus type 3 (PI-3) in ruminants in the Djibouti Republic. *Dtsch Tierarztl Wochenschr*. (1988) 95:99-102. https://pubmed.ncbi.nlm.nih.gov/2838248/
9. Brako E, Fulton R, Nicholson S, Amborski G, et al. Prevalence of bovine herpesvirus-1, bovine viral diarrhea, parainfluenza-3, goat respiratory syncytial, bovine leukemia, and bluetongue viral antibodies in sheep. *Am J Vet Res*. (1984) 45:813-6. https://pubmed.ncbi.nlm.nih.gov/6329044/
10. Bechmann G, Serological investigations in the diagnosis of viral infections derived from cattle in sheep. *Dtsch Tierarztl Wochenschr*. (1997) 104:321-1. https://pubmed.ncbi.nlm.nih.gov/9324461/
11. Schiefer P, Krametter-Frötscher R, Schleiner A, Loitsch A, Golja F, Möstl K, et al. Seroprevalence of antibodies to ruminant pestiviruses in sheep and goats in Tyrol (Austria). *Dtsch Tierarztl Wochenschr*. (2006) 113:55-8. https://pubmed.ncbi.nlm.nih.gov/16555484/
12. Preyler-Theiner B, Krametter-Frtscher R, Theiner A, et al. Studies on the seroprevalence of pestivirus infections in the goat population of Vorarlberg (Austria). *Wien Tierarztl Monat*. (2009) 96:232-9. doi: 10.1111/j.1532-950X.2008.00457.x

13. Molecular Characterization of Bovine Viral Diarrhea Virus Infection from Boer Goats.
14. Jetteur P, Thiry E, Pastoret PP. [Serological survey concerning the IBR, CHV2, BVD, PI3, BRS and rinderpest viruses in small ruminants in Zaire]. *Rev Elev Med Vet Pays Trop*. (1990) 43:435-7. https://pubmed.ncbi.nlm.nih.gov/2132781/
15. Krametter-Frtscher R, Loitsch A, Mstl K, Sommerfeld-Stur I, Baumgartner W. Seroprevalence of border disease and bovine viral diarrhoea in sheep and goats in selected regions of austria. *Wien Tierarztl Monat*. (2005) 92:238-44. https://www.researchgate.net/publication/286942653_Seroprevalence_of_Border_Disease_and_Bovine_Viral_Diarrhoea_in_sheep_and_goats_in_selected_regions_of_Austria
16. Adair BM, McFerran JB, McKillop ER, McCullough SJ. Survey for antibodies to respiratory viruses in two groups of sheep in Northern Ireland. *Vet Rec*. (1984) 115:403-6. doi: 10.1136/vr.115.16.403
**Reason 9: The type of sample collected is fecal sample (3 articles)**
1. Wang, Y.L., Xu, S.R., Zhang, X.Z., Xu, C.F. (2018). Etiological investigation and analysis of bovine viral diarrhea virus, border disease virus and enterovirus in Tibetan sheep in Haidong City, Qinghai Province. *Anim Husbandry & Vet Med* 50(12), 87-90. (In Chinese)
2. Wang, X.P.,Liu, H.,Xuan, H.,Zhu, W.Z., Ren, W.Z. (1993). Investigation of sheep infected with bovine viral diarrhea-mucosal disease virus. *CHIN J Vet Sci* 13(3), 219-221. doi: 10.16303/j.cnki.1005-4545.1993.03.003. (In Chinese)
3. Wang, X.P., Zhu, W.Z., Ren, W.Z., Cang, G.Q.,Hu, Q.X., Liu, H.Z.,et al. (1993) Investigation on Cattle and Sheep Infected with Viral Diarrhea-Mucous Disease.*CHIN J PREV VET MED* 15(4), 41-42. (In Chinese)

Figure S1. Egger's plot showing publication bias. (Immunological Methods)

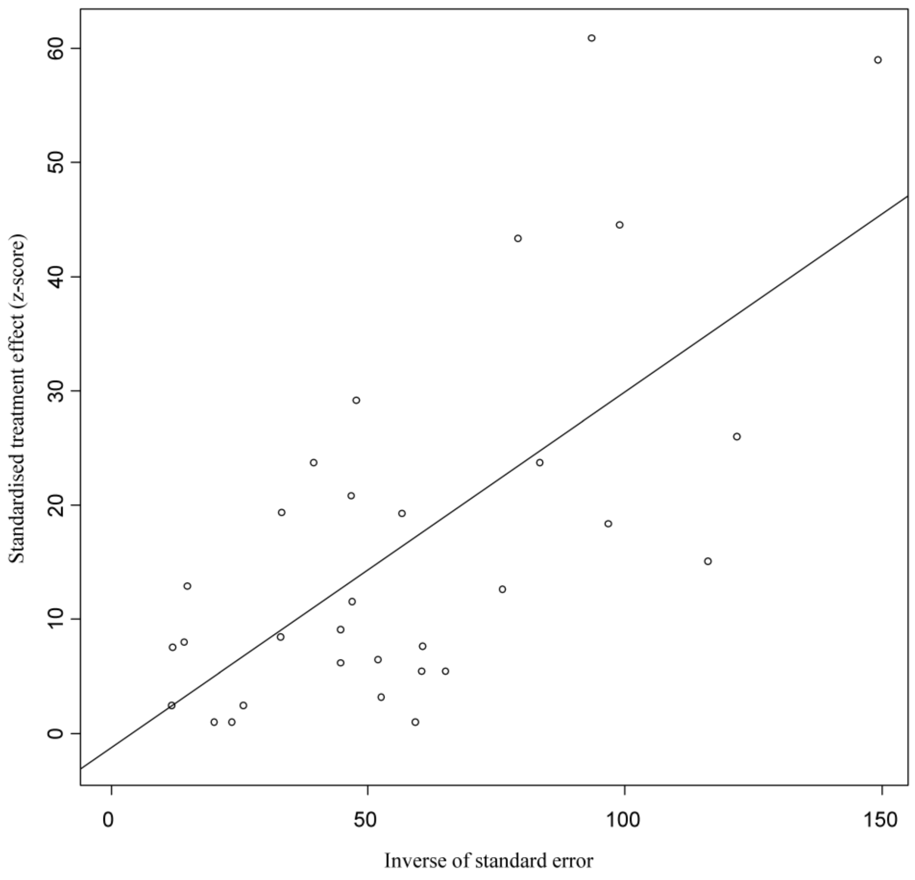

Figure S2. Egger's plot showing publication bias. (Molecular Methods)

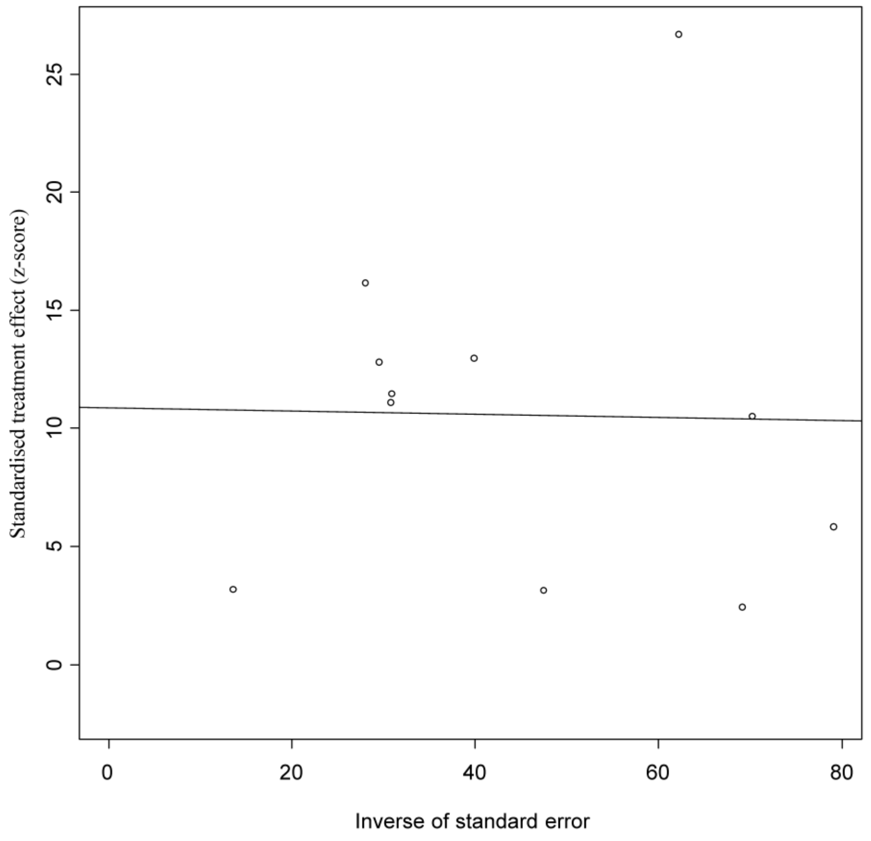

Figure S3. Publication bias of studies by trim and fill analysis. (Immunological Methods)

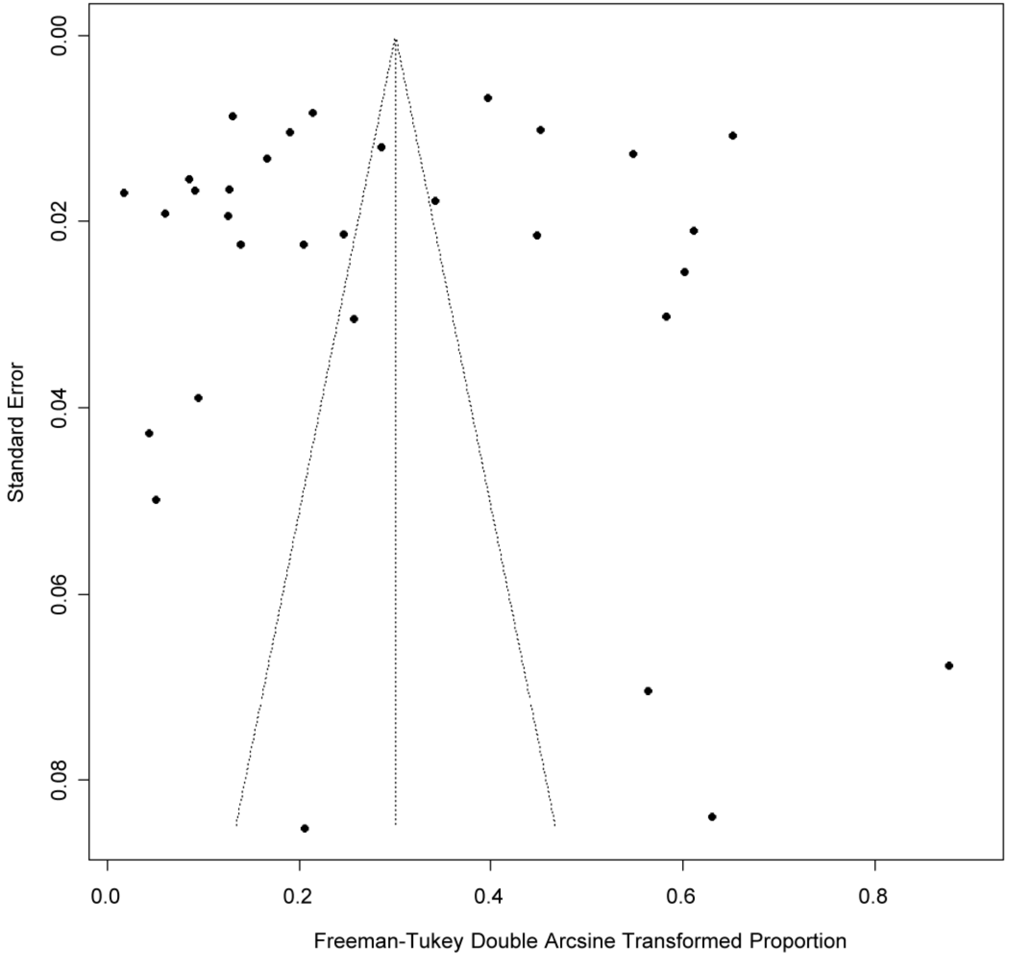

Figure S4. Publication bias of studies by trim and fill analysis. (Molecular Methods)

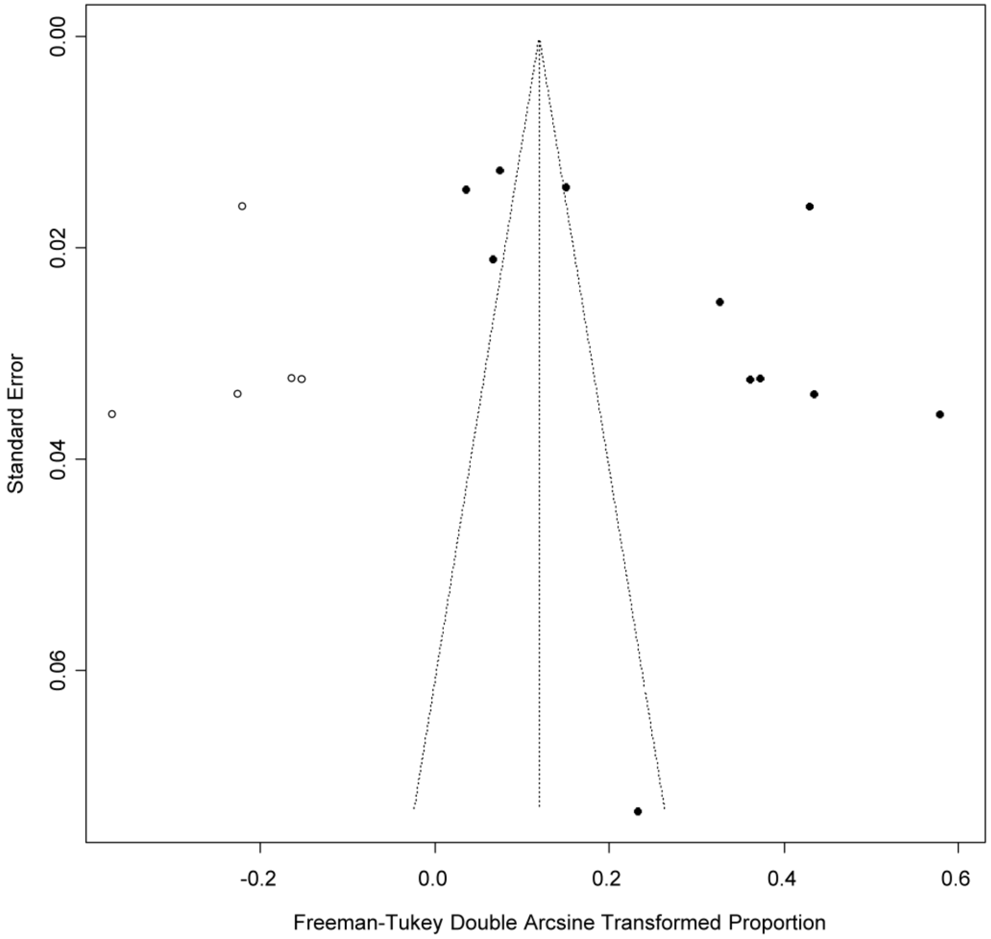

Figure S5. Sensitivity analysis. (Immunological Methods)

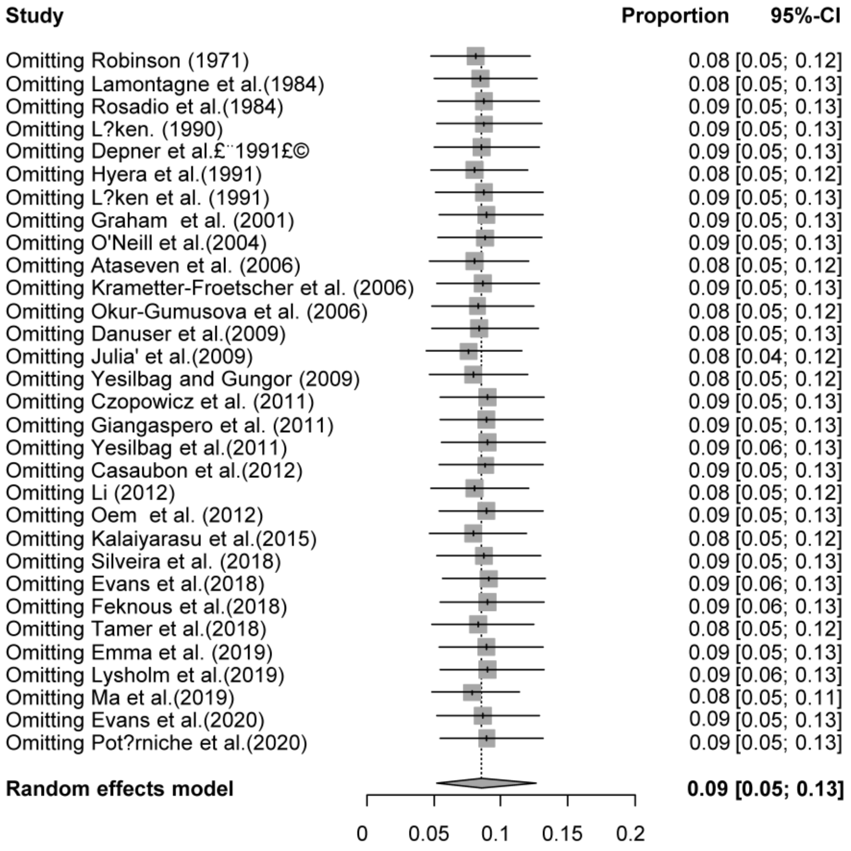

Figure S6. Sensitivity analysis. (Molecular Methods)

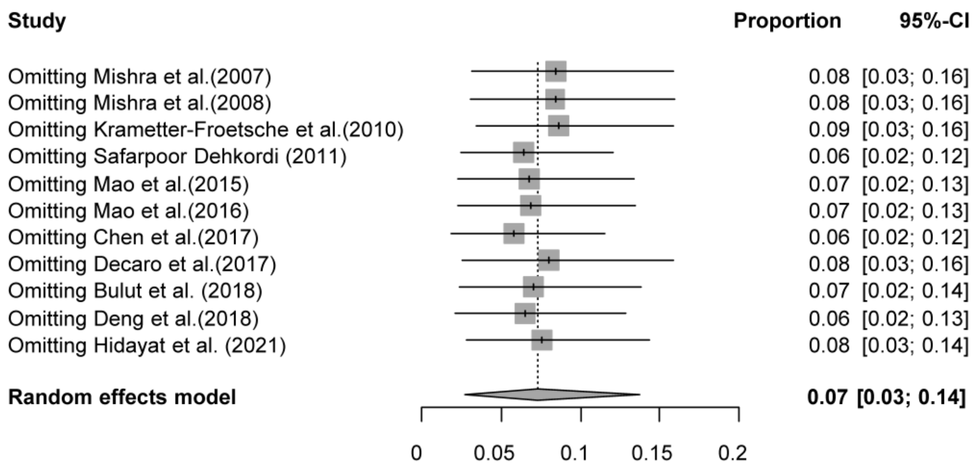

Supplement: Supplementary file 1 [file Data_Sheet_1.pdf]
